# Systems analysis of endothelial cell plasma membrane proteome of rat lung microvasculature

**DOI:** 10.1186/1477-5956-9-15

**Published:** 2011-03-29

**Authors:** Yan Li, Kerri Massey, Halina Witkiewicz, Jan E Schnitzer

**Affiliations:** 1 Proteogenomics Research Institute for Systems Medicine, 11107 Roselle Street, San Diego, California 92121, USA

## Abstract

**Background:**

Endothelial cells line all blood vessels to form the blood-tissue interface which is critical for maintaining organ homeostasis and facilitates molecular exchange. We recently used tissue subcellular fractionation combined with several multi-dimensional mass spectrometry-based techniques to enhance identification of lipid-embedded proteins for large-scale proteomic mapping of luminal endothelial cell plasma membranes isolated directly from rat lungs *in vivo*. The biological processes and functions of the proteins expressed at this important blood-tissue interface remain unexplored at a large scale.

**Results:**

We performed an unbiased systems analysis of the endothelial cell surface proteome containing over 1800 proteins to unravel the major functions and pathways apparent at this interface. As expected, many key functions of plasma membranes in general (i.e., cell surface signaling pathways, cytoskeletal organization, adhesion, membrane trafficking, metabolism, mechanotransduction, membrane fusion, and vesicle-mediated transport) and endothelial cells in particular (i.e., blood vessel development and maturation, angiogenesis, regulation of endothelial cell proliferation, protease activity, and endocytosis) were significantly overrepresented in this proteome. We found that endothelial cells express multiple proteins that mediate processes previously reported to be restricted to neuronal cells, such as neuronal survival and plasticity, axon growth and regeneration, synaptic vesicle trafficking and neurotransmitter metabolic process. Surprisingly, molecular machinery for protein synthesis was also detected as overrepresented, suggesting that endothelial cells, like neurons, can synthesize proteins locally at the cell surface.

**Conclusion:**

Our unbiased systems analysis has led to the potential discovery of unexpected functions in normal endothelium. The discovery of the existence of protein synthesis at the plasma membrane in endothelial cells provides new insight into the blood-tissue interface and endothelial cell surface biology.

## Introduction

The vascular endothelium forms a critical interface between blood and tissue. It largely controls the transfer of small and large molecules into and out of tissue. The vascular endothelium is highly specialized to meet the needs of the underlying tissue, and the phenotype and function of the vascular endothelium is dependent on the local microenvironment. As such, endothelial cells (ECs) from different tissues express different proteins and can even generate different responses to the same stimulus [1]. Though the vascular endothelium has been studied for decades, most of these studies have focused on a single protein or a single function. A global systems approach is needed to perform a holistic evaluation and reveal new functions and biological pathways of the endothelium.

Mass spectrometry (MS) has been a driving force propelling large-scale analysis of proteomes of complex biological samples. The advances in MS sensitivity, accuracy, robustness, ease-of-use and throughput have increasingly enabled researchers to identify and quantify proteins in tissues, cells, and subcellular compartments in depth. This has led to the discovery of biomarkers related to important processes and functions. Using MS technology, we have recently identified and quantified ~1800 proteins expressed at the luminal surface of EC plasma membranes (PM) isolated *in vivo *from rat lung vasculature [2,3]. However, global analyses are needed to move beyond a simple list of proteins and better understand how the component proteins interact and function in a given proteome. Ontology analysis, particularly at the systems level, can provide details of the biological and molecular functions associated with a given proteome and the molecular relationships within the proteome resulting in: 1) validation of a proteome generated from large-scale MS analysis; 2) insight into the relationship of proteins in a functional pathway; and 3) detection of functions and pathways novel to a given tissue, cell, or organelle.

Here, we used a previously acquired dataset obtained through MS analysis of the lung vascular endothelium to perform systems analysis and identify potentially novel endothelial and lung functions contained within this proteome. We systematically analyzed functional enrichment in our published lung EC PM membranome [2] using multiple public knowledge databases. We detected many major PM-associated functions highly enriched in the lung EC PM membranome as well as some unexpected, novel functions.

## Results

### Enrichment analysis of biological process in the EC PM membranome

The proteins expressed in a given tissue or cell likely define its functions under specific biological conditions. Thus, exploring biological functions associated with the proteins is essential to assess the fidelity of a proteome to its given tissue. We utilized three individual computational strategies, Avatar, FatiGO/GO, and IPA, to characterize the potential biological functions and pathways occurring in our rat lung EC PM membranome. Assignment of functions to each protein in Avatar included both manual article searches and importing annotations from two public databases, Panther ontology and UniProt knowledge database. To establish the relationship among all identified functions, we then used FatiGO algorithms to classify the functions of all proteins in this membranome and built the function-related tree structures based on the GO Tree Browser algorithms. The significance of enrichment of each functional category was determined by Z-score (see Materials and Methods).

**Figure 1:**
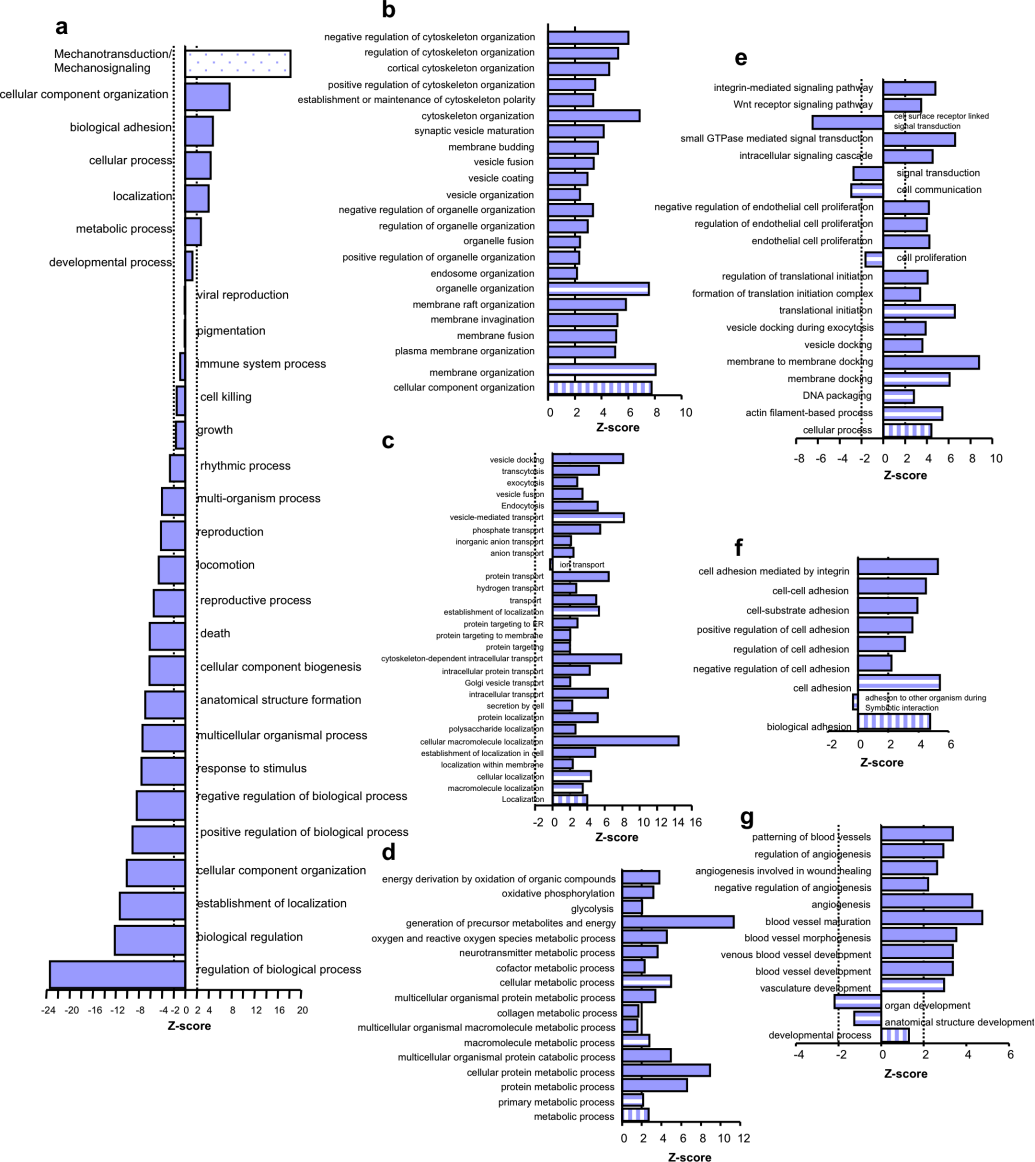
**Enriched biological processes in the membranome**. All proteins in the membranome were imported into FatiGO to search possible biological processes in the dataset using the rat knowledge database (see Materials and Methods). The over- or underrepresentation of each category was determined by a Z-score (≥2 or **≤-**2, dotted line(s) in the figure). All general categories in biological process included in this data are listed in (a). The subcategories under each general category are organized by GO algorithms and only selected categories are shown: cellular component organization (b), localization (c), metabolic process (d), cellular process (e), biological adhesion (f), and developmental process (g). The bars with vertical stripes indicate the main category from (a); the bars with horizontal stripes indicate the different subcategories in that functional unit; the solid bars are functional terms underneath the horizontal-striped bars, multiple levels can exist under the category of each horizontal-striped bar. Charts are arranged with the top level category at the bottom and resident subcategories above it.

Here, the results from the FatiGO/GO ontology analysis are presented; 1357 proteins (76%) of our dataset found matches in the FatiGO rat knowledge database. With respect to biological processes, 590 (43.5%) of the 1357 proteins were classified into 27 general biological processes (Figure 1a). We inserted mechanotransduction/mechanosignaling into this general group due to its significant enrichment in our membranome as per Avatar (Z > 18, see Materials and Methods) to yield 28 general categories as shown in figure 1a. In total, only 6 of the 28 general biological processes were significantly enriched in our dataset, consistent with the specialized nature of the membranome being analyzed. In addition to mechanotransduction/mechanosignaling, 5 GO categories were significantly overrepresented: cellular component organization (Z > 7), biological adhesion (Z > 4), cellular process (Z > 4), localization (Z > 4), and metabolic process (Z > 2).

Next, we further expanded general categories with positive Z-scores into their sublevels led by the GO Tree Browser. This allowed a more detailed analysis of the biological processes enriched at the EC PM (Figure 1b to 1g). With expansion of the most enriched GO category, cellular component organization, we discovered that only 2 of the 16 subcategories showed substantial enrichment: organelle organization (Z > 7) and membrane organization (Z > 8) (Figure 1b and Additional file 1, Figure S1). Further mining of membrane organization uncovered plasma membrane organization (Z > 5), membrane fusion (Z > 5), membrane invagination (Z > 5), and membrane raft organization (Z > 5) as enriched subcategories (Additional file 1, Figure S1). Vesicle coating (Z > 2), vesicle fusion (Z > 3), membrane budding (Z > 3), synaptic vesicle maturation (Z > 4), establishment or maintenance of cytoskeleton polarity (Z > 3), cortical cytoskeleton organization (Z > 4), and regulation of cytoskeleton organization (Z > 5) were significantly enriched subcategories of organelle organization (Additional file 1, Figure S1). All are consistent with expectations of a plasma membranome.

Similarly, we expanded another GO enriched category, localization. Three subcategories, macromolecular localization, cellular localization and establishment of localization were enriched. Further mining revealed that transport (Z > 5), protein localization (Z > 5), intracellular protein transport (Z > 4), and cytoskeleton-dependent intracellular transport (Z > 7) were the actual enriched processes under cellular localization and establishment of localization. By expanding the category of transport, we revealed that protein transport (Z > 6), hydrogen transport (Z > 2), exocytosis (Z > 2), vesicle fusion (Z > 3), endocytosis (Z > 5), transcytosis (Z > 5) and vesicle docking (Z > 8) were the actual enriched processes (Figure 1c and Additional file 1, Figure S2). More impressively, we found phosphate transport exhibited a significantly high Z-score (Z > 5) by mining two levels into the moderately depleted subcategory of ion transport (Z = -0.29) (Additional file 1, Figure S2). Also, phosphate transport was the only function that showed significant enrichment under the entire ion transport group. This suggests that substantial levels of energy metabolism and protein phosphorylation/dephosphorylation activities occur at the PM, in agreement with the fact that various transport and signaling transduction processes take place at the PM.

As indicated by the significant enrichment of the phosphate transport category in our membranome, energy metabolism may be enriched at the PM. To further explore this function, we expanded the general group of metabolic process. We found 19 subgroups in its next level, but only primary metabolic (Z > 2), macromolecule metabolic (Z > 2) and cellular metabolic processes (Z > 4) showed significant enrichment (Figure 1d, Additional file 1, Figure S3). Mining into these groups, we found that multicellular organismal protein catabolic process (Z > 4), multicellular organismal protein metabolic process (Z > 3), oxygen and reactive oxygen species metabolic process (Z > 5), oxidative phosphorylation (Z > 3), energy derivation by oxidation of organic compounds (Z > 4), and neurotransmitter metabolic process (Z > 4) were the actual enriched metabolic processes in this proteome (Figure 1d, Additional file 1, Figure S3). Enrichment in these categories is consistent with the enormous amount of receptor-related activities that occur for transport and signaling, vesicle-related activities, and protein secretion. Although finding neurotransmitter metabolic process as one of the overrepresented PM functions was initially surprising, ECs are known to express some classes of neurotransmitter receptors [4] and can possibly synthesize and release classic neurotransmitters [5]. Given that ECs and neurons share many morphological and developmental similarities, it is likely that these two cell types perform certain equivalent activities (see discussion). Thus, the enriched neurotransmitter metabolic process found in our work may be specific for only a few cell types (neurons and ECs), rather than processes that are enriched at the PM in general.

In part because the overrepresentation of phosphate transport in this proteome is consistent with signal transduction being enriched at the EC PM, we expanded both signaling transduction (Z = -2.7) and cell-cell signaling (Z = -0.12), subcategories under cell communication of the general category of cellular process, regardless of their Z-scores (Figure 1e, Additional file 1, Figure S4). Out of the over 30 expanded categories, only 3 subcategories of signal transduction were found significantly enriched: small GTPase-mediated signaling pathway (Z > 6), integrin-mediated signaling (Z > 4) and Wnt receptor signaling (Z > 3) (Figure 1e, Additional file 1, Figure S4). Furthermore, consistent with the enriched integrin-mediated signaling pathway found in the signal transduction subcategory, biological adhesion was one of the 5 enriched functions in biological processes (Figure 1a). Cell adhesion-mediated by integrin (Z > 5) was the most enriched subgroup in biological adhesion (Figure 1f).

Finally, to understand the role of developmental process (Z = 1.3) in the EC PM proteome, we mined the subcategory organ development (Z = -2.19), which is two levels down from development process. Surprisingly, vasculature development (Z = 3.0) was the only significantly enriched process out of the 24 organs in the list (Figure 1g and Additional file 1, Figure S5). Further mining of vasculature development revealed blood vessel development as significantly enriched. Under blood vessel development, venous blood vessel development (Z > 3), blood vessel maturation (Z > 4), and angiogenesis (Z > 4) (Figure 1g and Additional file 1, Figure S5) were enriched. These results are consistent with the endothelial source of the membrane proteome analyzed here. In addition, negative regulation of EC proliferation (Z > 4) was the only function that showed significant enrichment in the process of EC proliferation (Figure 1e and Additional file 1, Figure S4). This is compatible with our observation of blood vessel maturation being a significantly enriched process in our proteome, and the expectation that the normal endothelium is mature, and thus more quiescent, in the adult lung. It also suggests that the proliferation of ECs is dominantly controlled by inhibitory regulators (ATPase inhibitory factor 1, caveolin 1, and caveolin 2) to prevent growth under normal physiological conditions. Altogether, the significantly enriched processes within the main enriched biological processes are consistent with well-defined, fundamental roles of PM- and EC-associated proteins.

We were surprised to detect overrepresentation of certain processes, such as DNA packaging (Z = 2.8) and translational initiation (Z = 6.6) in the EC PM membranome (Figure 1e, Additional file 1, Figure S4). We investigated each protein identified in these categories using literature and database mining. We found that five out of the eight proteins in the DNA packaging category were either possibly associated with the PM or located at multiple subcellular compartments (Table 1). For instance, death-associated protein kinase 3, a nuclear protein, relocates to the cytoplasm and interacts with actin filaments [6]. If only the 3 proteins that appear to be specific to the nucleus were counted, DNA packaging would be underrepresented in our membranome (Z = 0.56). This suggests that the current databases are not fully annotated, due in part to the availability of the experimental data. Though all eight proteins were originally detected in the nucleus, it is likely that additional research will reveal that these proteins can translocate to other subcellular domains and even participate in entirely different functions. Although we could not exclude the activity of DNA packaging from the EC PM completely, it appears more likely that these proteins have secondary functions or were byproducts co-purified with the EC PM fractions.

**Table 1:** Proteins involved in DNA packaging.

JSID	Accession	Name	Subcellular specificity
JS40011197	O88807	Protein-arginine deiminase type-4	Cytoplasm

JS40071128	O88764	Death-associated protein kinase 3	Nuclear. Relocates to the cytoplasm.

JS40026124	Q923V8	15 kDa selenoprotein precursor	Endoplasmic reticulum lumen

JS40021817	Q63945	Protein SET [Phosphatase 2A inhibitor I2PP2A][I2PP2A][Template- activating factor I][TAF-I][Liver regeneration-related protein].	Cytoplasm, Endoplasmic reticulum, Nucleus

JS40021552	Q9Z2G8	Nucleosome assembly protein 1-like 1	Nucleus, Melanosome

JS40048134	NP_445899.1	Histone deacetylase 2	Nucleus

JS40016703	Q6AYU1	Mortality factor 4-like protein 1	Nucleus

JS40021952	Q6P747	Heterochromatin protein 1, binding protein 3	Nucleus

Proteins listed were taken from the FatiGO data sheet. From left to right, columns contain: Avatar ID, the accession number from RefSeq/UniProt database, the description of the protein, and the annotated subcellular location.

### Enrichment analysis of molecular function in the EC PM membranome

Having defined the biological processes presented in this proteome, we next decided to investigate the general activities these proteins are involved in, especially to reveal whether molecular activities correlate with the biological processes discussed above. As in the biological processes, we used FatiGO algorithms to classify the molecular functions of all proteins in this membranome and built the function-related tree structures based on the GO Tree Browser algorithms. The significance of enrichment of each functional category was determined by Z-score (see Materials and Methods).

**Figure 2:**
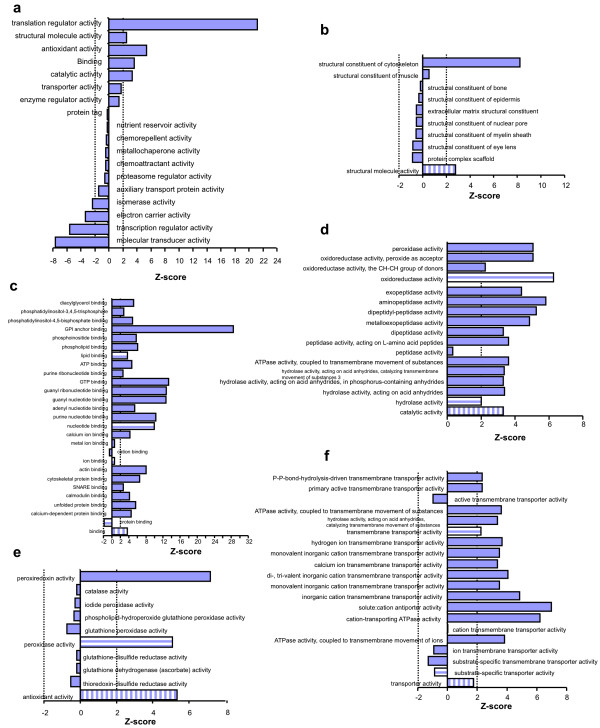
**Enriched molecular functions in the membranome**. All proteins in the membranome were imported into FatiGO to search for possible molecular functions in the dataset using the rat knowledge database (see Materials and Method). The over- or underrepresentation of each category was determined by a Z-score (≥2 or ≤-2, dotted line(s) in the figure). All general categories included in this data are listed in (a). The subcategories under each general category are organized by GO algorithms and only selected categories are shown: structural molecule activity (b), binding (c), catalytic activity (d), antioxidant activity (e), and transporter activity (f). The solid bars are functional terms under the horizontal-striped bars: see figure 1 for detail.

Using FatiGO/GO algorithms, we mapped 699 proteins (51.5% of the 1357) into 18 general categories (Figure 2a). Five of the eighteen general categories were significantly overrepresented in our dataset. Four of the five highly enriched functions can typically be associated with the PM, including structural molecule activity (Z > 2), binding (Z > 3), catalytic activity (Z > 3), and antioxidant activity (Z > 5). However, the most overrepresented molecular function in this proteome was translation regulator activity (Z > 22); it was surprising, but consistent with a similar finding in biological process (Figure 1e). We expanded the five significantly enriched molecular functions to reveal the exact molecular functions in the EC PM membranome.

In the category of structural molecule activity, the structural constituent of cytoskeleton (Z > 8) was the only overrepresented function within the 9 sub-level categories (Figure 2b). None of the other eight categories (protein complex scaffold, and structural constituent of eye lens, myelin sheath, nuclear port, extracellular matrix, epidermis and bone) were significantly enriched or depleted in the EC membranome. This is consistent with analysis of biological processes, which showed that cytoskeleton organization (Figure 1b) was also highly enriched (Figure 1b).

The general category of binding contained 38 subcategories; lipid binding (Z > 3) and nucleotide binding (Z > 9) were the only two significantly overrepresented functions. Further expanding these two groups, we found the actual enriched activities corresponded well with functions occurring primarily at the PM. For example, the extremely enriched GPI anchor binding (Z > 28), phosphatidylinositol-4,5-bisphosphate binding (Z > 4), and phosphatidylinositol-3,4,5-trisphosphate binding (Z > 2) correlated well with general signaling and phospholipid signaling pathways (Figure 2c and Additional file 1, Figure S6). It is also reassuring to see ATP binding (Z > 4) and GTP binding (Z > 13) being the definite enriched functions under nucleotide binding (Figure 2c, Additional file 1, Figure S6). Although the category of ion binding showed no enrichment at all (Z = 0.63), further mining revealed calcium ion binding activity was significantly enriched (Z > 4). This finding reinforced active transport and signaling pathways as being two significantly enriched biological processes of the EC PM membranome (Figure 1c &1e).

Expanding the enriched general category of catalytic activity revealed 19 subcategories. Oxidoreductase activity (Z > 6) was the most enriched subgroup (Figure 2d and Additional file 1, Figure S7). Expanding the oxidoreductase activity deeper resulted in recognizing peroxidase activity (Z > 5) as the actual enriched activity in this membranome (Figure 2d and Additional file 1, Figure S7). Peroxidase activity is also one of the subcategories in antioxidant activity (Figure 2e). With expansion of this category under antioxidant activity, we discovered the actual enriched activity was peroxiredoxin (Z > 7) (Figure 2e). Peroxiredoxins, a ubiquitous family of antioxidant proteins, play roles as both peroxidases and molecular chaperones by neutralizing reactive oxygen species (ROS) via catalyzing the formation of inter- or intra-molecular disulfide bonds. Overrepresentation of peroxiredoxins in this membranome may indicate the involvement of lung EC PM in 1) detoxification of ROS at the blood-tissue interface to protect underlying tissue from oxidation; 2) promotion of stress-induced chaperone activity at the PM via formation of higher molecular structures; and/or 3) modulation of the concentration of H_2_O_2 _at the PM for signal transduction [7].

The next most enriched function under catalytic activity was hydrolase activity (Z = 2.0) (Figure 2d and Additional file 1, Figure S7). Further mining revealed the most enriched subgroups were hydrolase activity, acting on acid anhydrides in phosphorus-containing anhydrides (Z > 3) and ATPase activity coupled to transmembrane movement of substances (namely ATP-binding cassette transporters, Z > 3) (Figure 2d, Additional file 1, Figure S7). Enrichment of these activities at the PM is logical and agrees well with the discovered enriched biological processes in the EC PM membranome, such as signal transduction, intracellular transport, vesicle-mediated transport, and transport of protein, hydrogen, and phosphate (Figure 1c &1e).

Peptidase activity is a well-described lung function [8,9]. In our previous work [10-12], we noticed an abundance of peptidases in our EC PM such as aminopeptidase P, aminopeptidase N, endothelin-converting enzyme, and angiotensin-converting enzyme. To better understand the role the lung EC PM plays in peptidase activity,, we mined peptidase activity further under catalytic activity in spite of its lower Z-score (Z = 0.34). As expected, we found metalloexopeptidase (Z > 3), dipeptidyl-peptidase (Z > 5), and aminopeptidase (Z > 5) significantly enriched in this membranome by mining into the exopeptidase activity (Figure 2d, Additional file 1, Figure S7). The over enrichment of exopeptidases in the EC PM highlights the possible role of generation of mature and/or active forms of membrane proteins by cleaving the N- and/or C-terminal sequences of the pro-protein at the cell surface, corresponds well with delivery of secreted proteins across the membrane, and suggests that PM proteins undergo active posttranslational modifications in the course of signal transduction and molecular transport.

Another key PM function is transporter activity. As discovered above, transport was one of the significantly enriched biological processes (Figure 1a). However, transporter activity (Z = 1.76) under molecular functions was not significantly enriched (Figure 2a). To seek the possible answer for the slight disagreement between these two types of annotations, we expanded transporter activity under molecular function. Remarkably, transmembrane transporter activity (Z > 2) was the only enriched activity among the 9 subcategories in this category, which explains the lower accumulated Z-score for the general category of transporter activity (Figure 2f, Additional file 1, Figure S8). Mining further into significantly enriched subcategories, we found that the actual overrepresented transmembrane transporter activities in the EC PM membranome were contributed by ATP-binding cassette transporters (Z > 3) and P-P-bond-hydrolysis-driven transmembrane transporter (Z > 2) (Figure 2f, Additional file 1, Figure S8). Both of these functions indicate that the major transporter activities in the EC PM membranome are energy-dependent transport of a solute. In addition, cation-transporting ATPase (Z > 6) and solute:cation antiporter activities (Z > 6) were also overrepresented under substrate-specific transporter activity (Figure 2f, Additional file 1, Figure S8). Moreover, the enriched hydrogen ion transmembrane transporter and calcium ion transmembrane transporter activities (Z > 3) (Figure 2f, Additional file 1, Figure S8) strongly supported the overrepresented hydrogen transport and signaling-related biological processes, as shown above (Figure 1c &1e). Taken together, this membrane is obviously highly involved in energy-dependent transmembrane transporter activities, more so than other types of transport, which are essential to regulate the intracellular milieu of EC.

**Figure 3:**
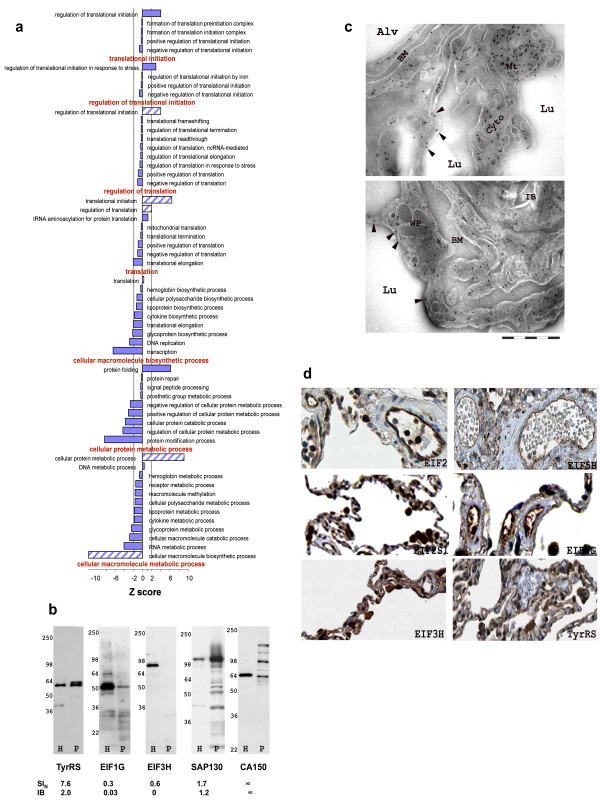
**Validation of protein synthesis at the EC PM**. The possibility of protein synthesis at the EC PM was investigated by: expansion of translation under biological process to reveal the final over enriched function in this membranome (a); immunoblot analysis to confirm molecules involved in translation in rat lung; H, total tissue homogenates; P, EC PM fraction (b); EM of rat lungs stained with anti-tyrosyl-tRNA synthetase (TyrRS) antibody and gold particles (c) (see Materials and Method). Top panel shows mitochondria (Mt), cytosol (Cyto), and plasma membrane (arrowhead) of endothelial cells (ECs) at the luminal surface of blood vessels. Bottom panel shows both endothelial and epithelial cells. Alv, alveolar; BM, basement membrane; Lu, capillary lumen; WP, Weibel-palade bodies in ECs; and IB, lamella body in epithelial cell. IHC of human normal lung vasculature from Human Protein Atlas (d) (see Materials and Methods). EIF1G, eukaryotic translation initiation factor 1 gamma; SAP130, splicing factor 3b, 130; CA150, transcription elongation factor 1; EIF3 H, eukaryotic translation initiation 3, subunit 9; EIF2, elongation factor 2; EIF5B, eukaryotic translation initiation factor 5B; EIF2S1, eukaryotic translation initiation factor 2, subunit 1 alpha; and TyrRS, Tyrosyl-tRNA synthetase.

To our surprise, translation regulator activity (Z > 22) was the most overrepresented function among the 18 general categories of molecular function in the EC PM membranome (Figure 2a). At this point, we reviewed all possible subcategories associated directly with protein synthesis based on GO algorithms to gauge the possible likelihood of protein synthesis at the PM. We sequentially mined into biological processes by expanding cellular macromolecule metabolic process and then cellular macromolecule biosynthetic process. We found translation (Z = 0.8) was not an enriched subcategory (Figure 3a, Additional file 1, Figure S3). However, further expanding, we discovered 2 functions: regulation of translation (Z > 2) and translation initiation (Z > 6), were significant enriched among the 8 subcategories in translation (Figure 3a). Mining into these two groups, we identified the actual enriched functions related to protein synthesis as regulation of translation initiation in response to stress (Z > 2) and regulation of translation initiation (Z > 3) (Figure 3a). In combination, both biological process and molecular function indicated that regulation of translation initiation, possibly in response to stress-related molecules, was of particular importance to the EC PM.

To further corroborate our intriguing finding of protein translation being present in the EC PM, we determined if any specific protein synthesis machinery was present in our membranome, especially initiation-related factors. Table 2 lists all translation initiation and elongation factors in our dataset. Of the 28 proteins we identified, only 15 had assigned functions based on the current knowledge database. Of these, nearly all annotated proteins (14) were cytoplasmic proteins. It is possible that some cytoplasmic proteins might randomly co-elute with the isolated EC PM fraction. However, if these proteins were merely contaminants, we would expect a more global representation of known cytosolic proteins. Instead, the abundance of these factors ranged from 5 to 18000 ng/mg (see Materials and Method); 2) their P/H ratios ranged from 0.07 to over 16 (see Materials and Method); and 3) of the 7 P-enriched (P/H > 1.0) factors detected in both H and P, all of them also showed high abundance in P (320 to 18000 ng/mg). The only exception was eukaryotic translation initiation factor 5B, which had a P/H > 4 but an abundance of <100 ng/mg. It is likely that some cytoplasmic proteins specifically and transiently interact with the PM. The presence on cytoplasmic proteins in our data set does not necessarily indicate contamination. Indeed, the varied abundance and enrichment of these proteins suggests a specific interaction rather than general contamination.

**Table 2:** Proteins involved in regulation of translational initiation and elongation found in the EC PM membranome.

JSID	Accession	Description	Ave ng/mg membrane	P/H	Location	IHC (HPA)
JS40004190	NP_787032	eukaryotic translation elongation factor 1 alpha 1	18258.65	2.56	Cytoplasm	NF

JS40005734	NP_001100433	eukaryotic translation initiation factor 1A, Y-linked	2626.9	10.755	undecided	minimal stain

JS40005646	NP_062229	eukaryotic translation initiation factor 2, subunit 1 alpha	2308.39	6.771	undecided	vessels

JS40001804	NP_001094012	eukaryotic translation initiation factor 2, subunit 3	775.64	16.697	undecided	no stain on vessels

JS40006902	NP_955412	Eukaryotic translation initiation factor 2, subunit 2 beta, 38 kDa.	763.77	5.164	undecided	negative

JS40001854	NP_001102719	eukaryotic elongation factor, selenocysteine-tRNA-specific	358.95	**∞**	Cytoplasm; Nuclear	negative

JS40020543	EDM15826	eukaryotic translation initiation factor 3, subunit 6 interacting protein (Eukaryotic translation initiation factor 3 subunit L)	317.75	1.972	Cytoplasm cytoplasm; ER;	negative no stain on

JS40021548	XP_226974	PREDICTED: similar to eukaryotic translation initiation factor 5A2	267.31	0.258	cytoplasm; ER;	no stain on

JS40011343	NP_001099762	eukaryotic translation initiation factor 3, subunit F	232.31	0.329	Cytoplasm	NT

JS50132783	NP_001032429	eukaryotic translation initiation factor 6	228.67	**∞**	Cytoplasm; Nucleus	NT

JS40017299	NP_001102269	Eukaryotic translation elongation factor 1 beta 2	147.59	0.922	undecided	NT

JS40009434	P05197	Elongation factor 2.	125.01	0.079	Cytoplasm	vessels

JS40023935	Q07205	Eukaryotic translation initiation factor 5.	97.1	**∞**	undecided	minimal stain

JS40005388	NP_001103611	eukaryotic translation initiation factor 5B	96.07	4.506	Cytoplasm	vessels no stain on

JS40033059	NP_001040552	eukaryotic translation initiation factor 3 subunit A	92.23	0.59	Cytoplasm	vessels

JS40021779	NP_001099712	eukaryotic translation initiation factor 3, subunit K	74.25	**∞**	Cytoplasm; nucleus	NT

JS40017420	NP_001004223	eukaryotic translation elongation factor 1 gamma	73.2	0.307	undecided	vessels

JS40038166	Q63186	Translation initiation factor eIF-2B subunit gamma	35.96	**∞**	undecided	no stain on vessels

JS40041107	Q64270	Translation initiation factor eIF-2B subunit alpha	34.34	**∞**	undecided	NT

JS50104474	Q4G061	Eukaryotic translation initiation factor 3 subunit B	29.64	0.139	Cytoplasm	no stain on vessels

JS40046084	NP_001013122	Eukaryotic translation elongation factor 1 delta.	24.97	0.293	undecided	NT

JS40032823	Q8VHU4	Elongator complex protein 1	14.96	**∞**	cytoplasm; nucleus	NF

JS40020615	NP_001099307	eukaryotic translation initiation factor 1	14.26	**∞**	undecided	NT

JS50042945	Q3B8Q2	Eukaryotic initiation factor 4A-III	11.75	0.304	cytoplasm; nucleus	no stain on vessels vessels

JS40069686	NP_001099765	Tu translation elongation factor, mitochondrial	8.17	0.07	Mitochondria	minimal stain

JS40039662	Q6P9U8	Eukaryotic translation initiation factor 3, subunit 3 gamma, 40 kDa.	7.31	0.18	Cytoplasm	vessels

JS40041278	P70541	Translation initiation factor eIF-2B subunit delta	7.08	**∞**	undecided	minimal stain

JS40053968	NP_001102809	eukaryotic translation initiation factor 2A	4.9	**∞**	undecided	negative

NF: Not Found in HPA; NT: Not Tested by HPA

Protein listed were taken from our rat lung EC PM proteome study published previously [2]. From left to right, columns contain: Avatar ID, the accession number from the RefSeq database, description of the protein, abundance, P/H ratio, annotated subcellular location, and status of IHC analysis in HPA.

Next, we performed Western analysis of H and P to validate our MS data and to better establish the presence of proteins related to protein synthesis in P. Several antibodies obtained showed poor reactivity or specificity. Only 5 worked and 4 of them confirmed expressions in P (Figure 3b), thereby supporting the potential existence of protein synthesis machinery in our membranome. For example, Tyrosyl-tRNA synthetase (TyrRS) and splicing factor 3b, subunit 3 (SAP130) showed much stronger signal in P than in H, plus their P/H ratios determined through densitometry analysis of the immunoblot correlated closely with the P/H ratios generated through MS analysis (Figure 3b).

Immunoblotting P revealed three very distinct bands for transcription elongation regulator 1 (CA150) with molecular weights ranging from 66 to 120 KDa, but only the band with the lowest molecular weight was detected in H (Figure 3b). These three bands in P may correspond to multiple splice isoforms of this transcription factor found in mouse and *Schistosoma mansoni *[13,14]. CA150 is currently known as a nuclear protein [15]; however, the distinct distribution pattern of its isoforms in our EC PM suggested that the protein may have multiple locations and functions, some of which may be unique to ECs. These isoforms could exist in different subcellular compartments like the PM to mediate distinct functions.

Two translational initiation elongation factors exhibited strong signals in H but exhibited fairly weak (EIF1G) or no signal (EIF3H) in P by immunoblotting (Figure 3b). These results agreed with the SI_N _values and P/H ratios derived from our MS results, which showed both proteins were underrepresented in P (P/H, 0.3 and 0.6).

We then investigated the actual subcellular location of these proteins using immuno-electron microscopy (EM) of rat lungs. Only one antibody, anti-TyrRS, worked by immuno-EM and revealed protein located in the cytoplasm, PM, and mitochondria of ECs in rat lung (Figure 3c). TyrRS expression in mitochondria [16] and in the cytoplasm [17] has been reported. To our knowledge, localization at the EC PM is a novel finding.

For further assessment of PM localization of protein synthesis machinery at the EC surface, we searched the IHC database of the Human Protein Atlas (HPA, http://www.proteinatlas.org) for positive staining of candidate proteins on normal lung vasculature. Candidate proteins included all initiation factors listed in Table 2, the two splicing factors (CA150 and SAP130) and TyrRS, all of which showed positive signals in our P by Western blot. In this search, we found 19 out of the 28 translation initiation factors had been tested by IHC analysis. Antibodies to 15 of these proteins showed positive signal in the lung tissues; only 9 showed lung vessel immunostaining (Table 2). Five of the 9 translation initiation factors that were localized to lung vessels were present at relatively high levels at the surface of the vasculature (Figure 3d), whereas expression of 4 proteins appeared more moderate (data not shown). Neither of the two splicing factors was tested by IHC in the current HPA. TyrRS also showed very nice signals on the surface of the vessels in lungs. Cumulatively, it appears that eukaryotic translation elongation factor 1 gamma (EIF1G) and TyrRS are indeed located at the EC PM according to our immunoblotting, EM and IHC of Human Protein Atlas.

### Interaction network analysis of EC PM

Instead of acting alone, proteins in cells form complexes by interacting with each other in order to exert their functions effectively and efficiently. Therefore, in theory, proteins in a "subproteome" should display apparent function-dependent relationships. To determine whether the proteins in our membranome form functional units, we input all proteins identified in the EC PM membranome into Ingenuity Pathway Analysis (IPA), a protein network and pathway analysis algorithm. Over 1500 proteins found matches in the IPA knowledge database (IPAKDB), of which 669 were eligible for network analysis and 674 were eligible for pathway/function analysis. These molecules were distributed into 90 networks when only direct protein-protein interactions were examined using the IPA algorithm, of which 32 networks had scores ≥ 10 (Table 3, see Materials and Methods). The IPAKDB defines direct protein-protein interactions through annotations from the literature. Not all direct interactions have been extensively proven, and these interactions must be used with caution. Consistent with our ontology analysis, cell morphology, cellular development, cellular function and maintenance, transport, cellular assembly and organization, cardiovascular system development and function, cell signaling, and protein synthesis were among the top 10 networks.

**Table 3:** Top-ranked protein-protein interaction networks in IPA.

ID	Top Functions	Score	Focus Molecules
1	Cell Morphology, Cellular Development, Cellular Function and Maintenance	46	33

2	Lipid Metabolism, Molecular Transport, Small Molecule Biochemistry	39	30

3	Cellular Assembly and Organization, Cancer, Reproductive System Disease	39	30

4	Cancer, Reproductive System Disease, Cardiovascular System Development and Function	37	29

5	Gene Expression, Cell Cycle, Cancer	35	28

6	Cell-To-Cell Signaling and Interaction, Immune Response, Cellular Development	33	27

7	Inflammatory Disease, Respiratory Disease, Protein Synthesis	33	27

8	Cell Signaling, Vitamin and Mineral Metabolism, Cancer	27	24

9	Cellular Assembly and Organization, Cellular Compromise, Cell Morphology	27	24

10	Cell Signaling, Connective Tissue Disorders, Dermatological Diseases and Conditions	25	23

11	Lipid Metabolism, Nucleic Acid Metabolism, Small Molecule Biochemistry	20	20

12	Cell-To-Cell Signaling and Interaction, Tissue Development, Cancer	19	19

13	Cellular Development, Cell Morphology, Respiratory Disease	17	18

14	Cancer, Cellular Movement, Cell Morphology	16	17

15	Neurological Disease, Cell Cycle, Genetic Disorder	16	17

16	Cancer, Cellular Movement, Cell Morphology	16	17

17	Amino Acid Metabolism, Post-Translational Modification, Small Molecule Biochemistry	16	17

18	Genetic Disorder, Neurological Disease, Protein Synthesis	16	17

19	Cancer, Cell Morphology, Hematological Disease	15	14

20	Post-Translational Modification, Protein Folding, Molecular Transport	14	16

21	Cell Death, Neurological Disease, Cancer	14	16

22	Cellular Function and Maintenance, Small Molecule Biochemistry, Cell Cycle	14	16

23	Cell Signaling, Cell Morphology, Cellular Function and Maintenance	14	16

24	Gene Expression, Cell-To-Cell Signaling and Interaction, Renal and Urological System Development and Function	13	14

25	DNA Replication, Recombination, and Repair, Nucleic Acid Metabolism, Small Molecule Biochemistry	13	15

26	Cellular Assembly and Organization, Cell Signaling, Cellular Function and Maintenance	13	15

27	Free Radical Scavenging, Cell-To-Cell Signaling and Interaction, Cell Morphology	13	15

28	Cellular Function and Maintenance, Cell Signaling, Molecular Transport	13	15

29	Gene Expression, RNA Post-Transcriptional Modification, Cancer	13	15

30	Cell Cycle, Tissue Development, Gene Expression	12	14

31	Cellular Development, Nervous System Development and Function, Tissue Morphology	12	14

32	Lipid Metabolism, Molecular Transport, Small Molecule Biochemistry	10	13

This table lists all networks having a score ≥10 from the IPA algorithms (see Materials and Methods). From the left to right, ranks from IPA, functions in that rank, score, and the number of molecules from our dataset in that group.

**Figure 4:**
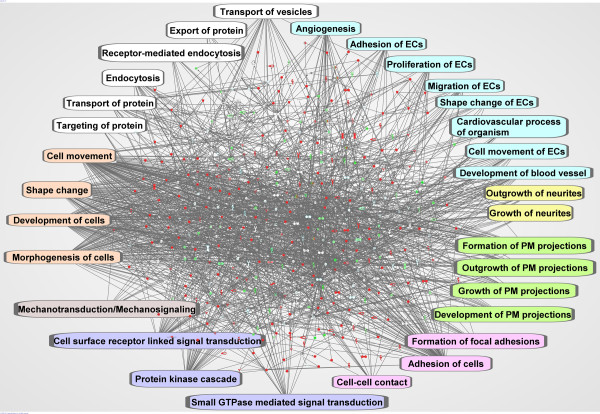
**Global protein-protein interaction network of the EC PM membranome**. All proteins in the EC PM membranome were imported into IPA (see Materials and Methods). Molecules found within the IPA knowledge database (IPAKDB) were applied to the network analysis and only direct relationships among the molecules were allowed. The top 14 networks were merged to create this global map. Known PM- or EC-associated functions are highlighted with blue for EC-associated functions; yellow for neuron-associated functions; green for PM-associated functions; pink for adhesion-associated functions; purple for signaling pathways; light pink for mechanotransduction/mechanosignaling; orange for cell movement and morphology; and white for transport and trafficking. Red nodes indicate enriched proteins in the PM (P/H > 1.0). Green nodes indicate the depletion of the protein in P (P/H < 1.0). The darker the red/green is, the greater the enrichment or depletion. White nodes indicate the proteins were not found in the membranome.

We merged the top 14 networks to build a general interactome of the EC PM membranome to examine cross-relationships among the molecules in the top-ranked networks (Figure 4). This global map consisted of 491 nodes and 2292 edges, reaching an edge to node ratio of >4, indicating extremely strong interaction among the molecules in this network. Using the p-value provided by IPA (see Materials and Methods), in comparison of our dataset with IPAKB, many PM- or EC-related functions were significantly overrepresented in this global map (4.6E-5>p > 3.2E-15) including formation of PM projections, cell surface receptor linked signal transduction, proliferation of ECs, angiogenesis and protein trafficking. Moreover, the most significantly enriched functions were cellular movement, cell morphology, cellular development, cell-to-cell signaling, cell-to-cell interaction and tissue development (5.7E-22>p > 4.6E-32, see Materials and Methods).

To explore the detailed molecular relationships in the global map, we expanded this map, particularly the regions highly relevant to functions at the PM that were also identified in our ontology analyses. We extracted all molecules assigned by IPAKDB from the global map, then manually imported the molecules in both our dataset and IPAKDB but absent in the global map. Still, we permitted only direct interactions between molecules in these expanded networks.

**Figure 5:**
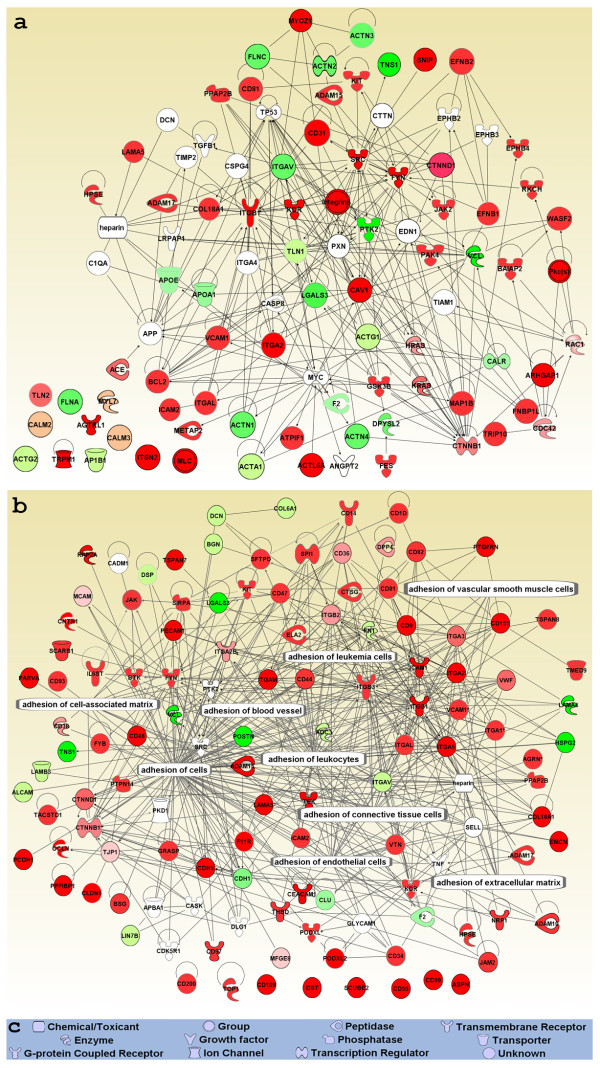
**Expanded networks of mechanotransduction/mechanosignaling and adhesion**. All molecules associated with each function were taken from both figure 4 and our membranome if they were not included in the top 14 networks. Protein-protein associations are indicated by single lines, whereas the arrows indicate the protein controls the other's expression. Only direct protein-protein interaction was allowed in these detailed networks, Mechanotransduction/mechanosignaling (a) and adhesion (b). Each node shape represents a different class of molecule, as shown in (c). Red nodes show that the proteins were enriched in the PM (P/H > 1.0). Green nodes indicate the depletion of the protein in P (P/H < 1.0). The darker the colors are, the greater the enrichment or depletion. White nodes indicate the proteins were not found in our dataset.

The network of mechanotransduction/mechanosignaling was generated by overlaying all assigned molecules, identified per Avatar, onto the global interactome. To create the final map, we extracted molecules from the global interactome and manually added the molecules in our data set annotated as mechanotransduction/mechanosignaling but not originally listed in the global interactome. Using IPA algorithms, 95 nodes were connected by 293 edges in the mechanotransduction/mechanosignaling group (Figure 5a). The edge to node ratio of >3.0 clearly indicated that molecules in this group definitely interacted with each other to function as a unit. Not surprisingly, not all proteins in this interactome were found in the EC PM data set. Such as, 18 proteins were not found in our dataset; 7 were involved in this network (Figure 5a). Of these, 2 were nuclear proteins [MYC (v-myc myelocytomatosis viral oncogene homolog) and CASP8 (caspase 8)]; 1 was cytoplasmic protein [TIAM1 (T-cell lymphoma invasion and metastasis 1)]; 1 was a plasma membrane protein [ITGA4 (integrin, alpha 4)]; and 3 were multiple location proteins [PXN (paxillin), TP53 (tumor protein p53), and APP (amyloid beta A4 precursor protein)]. These proteins were also not listed in our curated category of mechanotransduction/mechanosignaling. Likewise, two ephrin receptors, EPHB2 and EPHB3 were missing from both our dataset and the list of mechanotransduction/mechanosignaling proteins. Lack of these proteins in our list may be caused by limited database and article searches or their novelty to this network, or may represent downstream signaling molecules. Eleven molecules from our dataset showed no relationship with any other molecules in this group, suggesting further research of both the biological processes and molecular functions of these molecules is required to evaluate whether they should be incorporated in this network.

The interactome for adhesion was generated by using molecules in the global map related to adhesion and manual addition of the molecules absent from the global map but annotated as adhesion in our dataset, as per Avatar (Figure 5b). This network contained 121 nodes and 390 edges, giving rise to an edge to node ratio of 3.2, indicating very strong relationships among these molecules.

**Figure 6:**
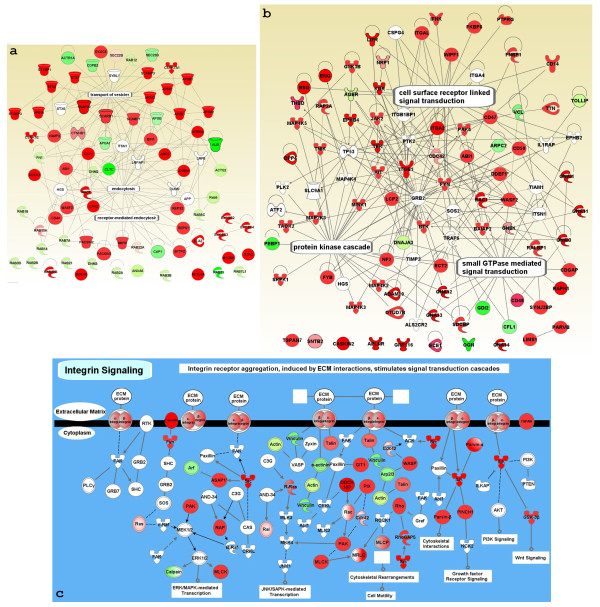
**Expanded networks of transport and signaling pathways**. The relationships among the molecules involved in transport (a) and signaling pathways (b) are graphed as described in the text. Integrin-mediated signaling pathway derived by the canonical pathway algorithms of IPA (c). The node colors and shapes are described in figure 5.

Transport and signaling are another two key functions of the PM. Since they are diversified functional groups, these interactomes were built by using the molecules extracted from the global map only. Molecules in the groups highlighted in white shown in figure 4 were used to create the transport network resulting in a network of 77 nodes and 138 edges. Fourteen molecules lacked connections in this map, which may have been caused by the loss of interacting molecules after extraction (Figure 6a). The majority of the molecules in this network were involved in endocytosis, consistent with the presence of transport vesicles such as caveolae on EC surface membranes.

The molecules in the integrin-mediated signaling pathway, small GTPase-mediated signaling transduction, and protein kinase cascade in figure 4 were extracted to build the signaling pathway interactome where 97 nodes were linked by 212 edges leading to an average of >2 edges per node (Figure 6b). This network demonstrated that the molecules in these three pathways not only interacted with each other, but also shared many common proteins. When analyzing the integrin-mediated signaling pathway with the IPA pathway algorithms, we discovered cell motility resulting from cytoskeletal rearrangement was the dominant pathway induced by integrin-mediated signaling in this membranome (Figure 6c). It was consistent with the finding of the highly overrepresented cytoskeleton organization (Z > 8) in our ontology analysis (Figure 1b and Additional file 1, Figure S1). Hence, the data from IPA agreed well with our previous analyses and, more importantly, highlighted the functions of the PM (Table 3 and Figure 1b).

Based on the endothelial nature of the PM analyzed, we expected to identify EC-related functions associated tightly with each other. Consistent with the ontology analyses, we detected a number of vasculature development-linked biological processes using IPA algorithms, such as angiogenesis, development of blood vessel, cardiovascular process of organism, EC proliferation, EC migration, EC movement, EC shape change, and EC adhesion (Figure 4). To view the detailed relationship between these molecules, we extracted all molecules in these groups and built two extended interactomes, angiogenesis and EC-related functions.

**Figure 7:**
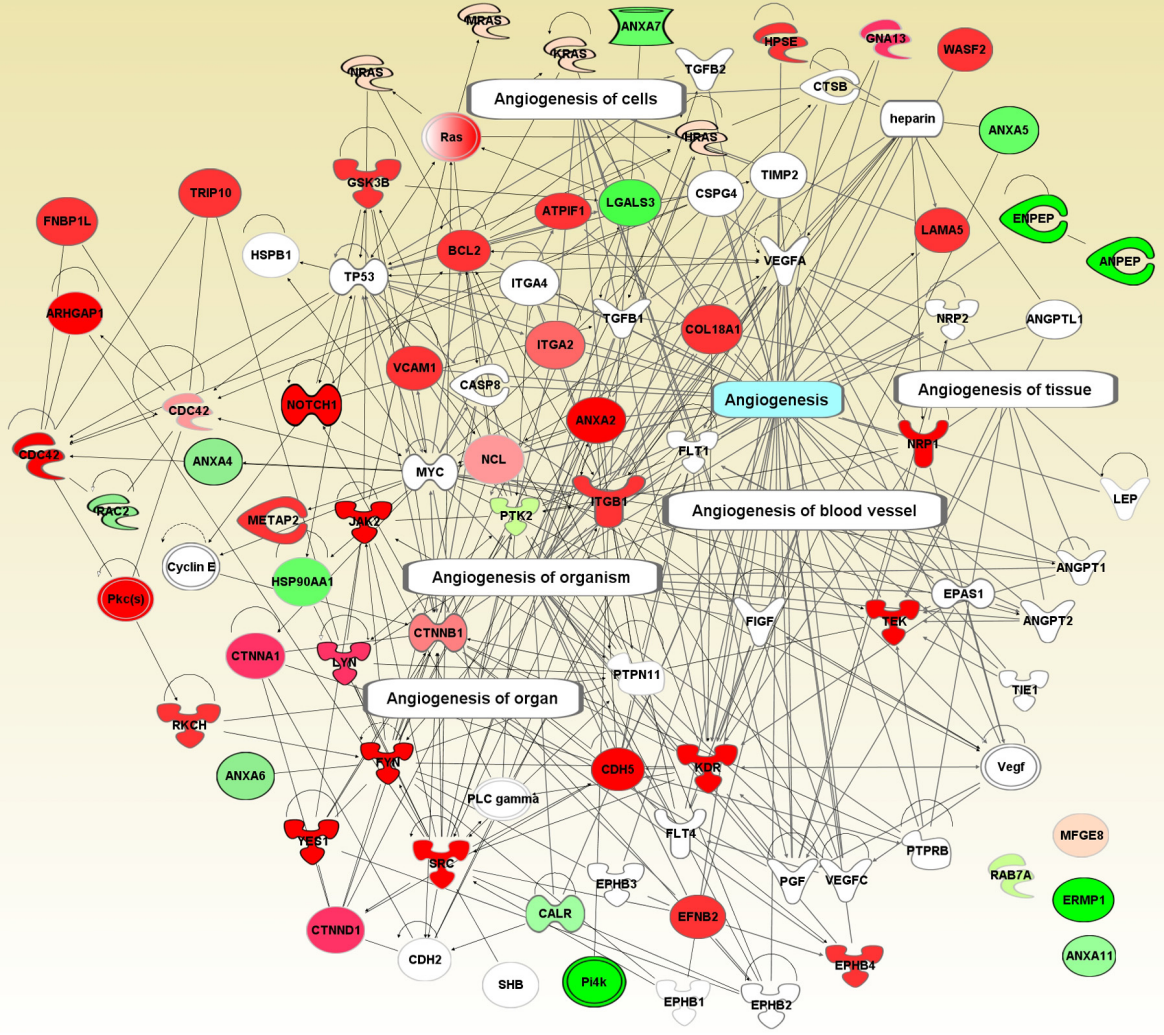
**Network of angiogenesis**. This network was created with molecules marked as angiogenesis in figure 4 and molecules in Avatar annotated as angiogenesis but not initially in figure 4. Only direct protein-protein interactions were allowed. See figure 5 for the colors and shapes of the nodes.

The angiogenesis interactome was constructed by extracting molecules from the global map of angiogenesis and adding molecules in our dataset as per Avatar (see Materials and Methods). This network was built upon 91 nodes and 313 edges resulting in >3 edges per node, except for 4 molecules (Figure 7). Annexin A11 (ANXA11), Rab-7A, endoplasmic reticulum metallopeptidase 1 (ERMP1), and milk fat globule-EGF factor 8 (MFGE8) were annotated as angiogenesis molecules but showed no direct interactions with any other molecules in this network. It is possible that this exclusion may be caused by missing molecules with which they directly interacted in our dataset or by the limitations of the IPAKDB.

**Figure 8:**
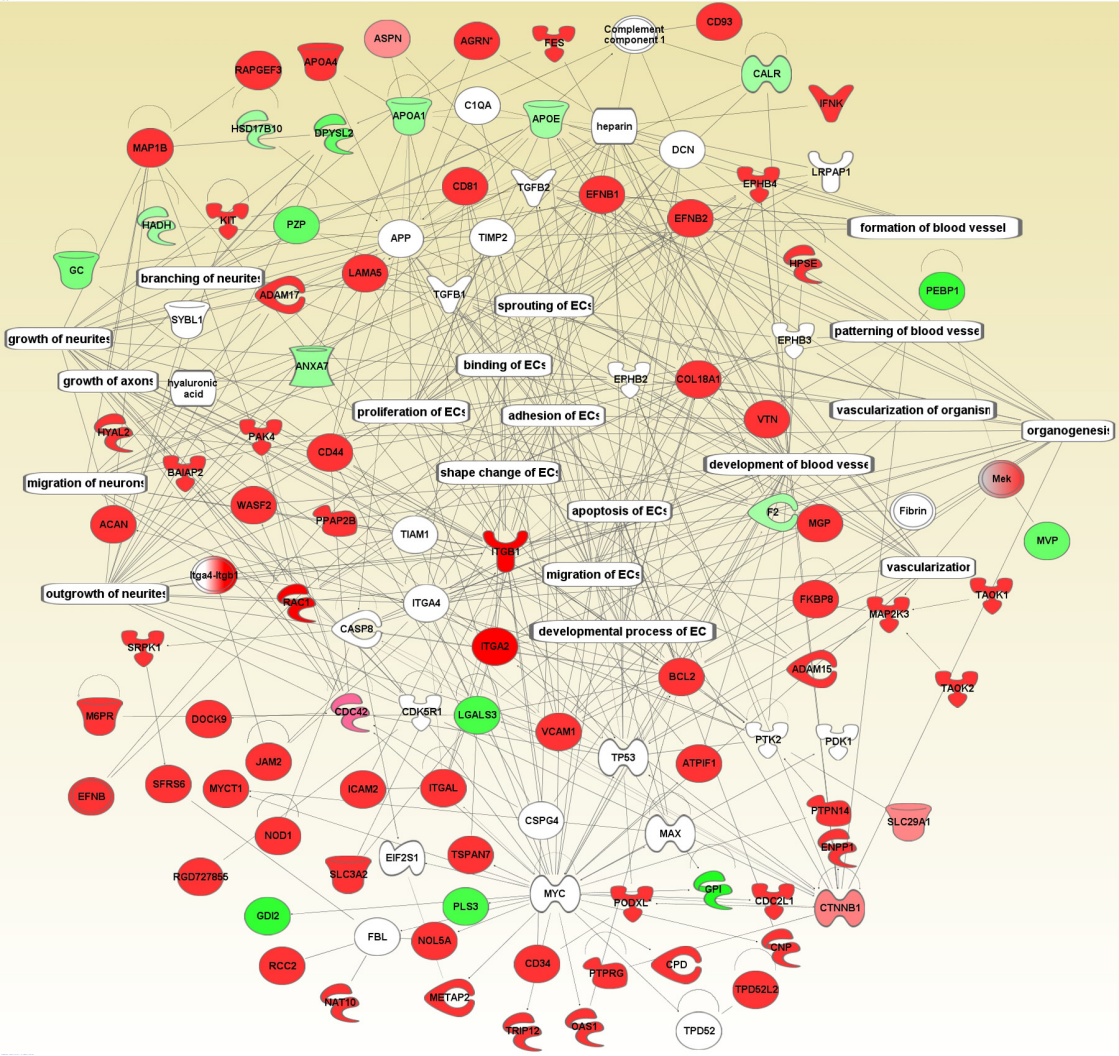
**Network of EC-associated functions**. As in figure 7, all molecules associated with EC-related functions and growth of neurites from figure 4 were extracted to generate this network. Only direct protein-protein interactions were allowed in the analysis. See figure 5 for the node colors and shapes.

The interactome for EC-related function was generated by directly connecting all molecules associated with EC activities and two neurite activities, outgrowth of neurites and growth of neurites (Figure 4). This map consisted of 113 nodes and 249 edges reaching an edge/node ratio of >2, indicating these molecules worked together as a functional entity (Figure 8). It is worth mentioning that every input molecule found partners to interact with. Additionally, the majority of molecules from our dataset (63 out of 76) were highly enriched in the P fraction (P/H > 1.0, the red nodes).

**Figure 9:**
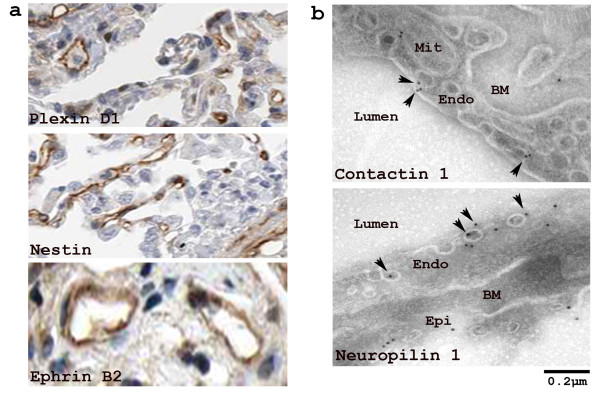
**Validation of neuron-restricted proteins at the EC PM**. The possibility of the neuron-restricted proteins at the EC PM was investigated by: IHC analysis of human normal lung vasculature from Human Protein Atlas with plexin D1 as positive control (a) and EM of rat lungs stained with anti-contactin 1 (top) or anti-neuropilin 1 (bottom) antibody and gold particles (b) (see Materials and Method). Subcellular locations of mitochondrion (Mit), endothelium (Endo), basement membrane (BM), epithelium (Epi), and plasma membrane (arrowhead) of endothelial cells (ECs) at the luminal surface of blood vessels are as indicated in each image.

The presence of these two neurite activities in our EC PM proteome and their strong molecular relationship with EC-related functions led us to investigate other neuron-related proteins in our proteome. We found 14 proteins that are thought to be primarily neuron-associated and even neuron-restricted (Table 4), including trafficking proteins (VAMPs, reticulon-1 and neurobeachin) [18-20]; adhesion and structural proteins (N-catenin, contactin, shank and N-wash) [21-24]; and growth and differentiation proteins (neuropilin, BAI-associated protein 2, plexin B, reticulons and nestin) [18,25-30]. The abundance and the enrichment of the proteins in this group ranged from 1 to >600 ng/mg with P/H ratio from 0.5 to 5. Four proteins in this group have been further confirmed as present at the EC PM by either IHC (Figure 9a) or EM (Figure 9b).

**Table 4:** Proteins involved in neuron-restricted processes found in the EC PM membranome.

JSID	Accession Number	Description	Quantity (ng/mg membrane)	P/H	Location	Tissue specificity (UniProt & GO)
JS40001915	Q9Z270	Vesicle-associated membrane protein-associated protein A [19]	645.54	0.523	Plasma membrane; vesicle	Ubiquitous

JS40008100	NP_001100068	catenin (cadherin associated protein), alpha 2 (N-catenin) [24]	461.25	3.92	plasma membrane, Cytoskeleton, cell junctioin	Brain

JS40003261	Q9JK11	Reticulon-4 (nogo) [18]	343.33	1.09	Endoplasmic reticulum	isoform 1 is expressed mainly in the nervous system, Isoforms 2 and 3 are detected in other tissues other than CNS

JS40015951	Q9Z269	Vesicle-associated membrane protein-associated protein B [19]	267.42	0.461	Plasma membrane; vesicle	Ubiquitous

JS40003589	Q9QWJ9	Neuropilin-1, CD304 [29]	218.03	2.342	Plasma membrane	Neuron and EC

JS40013915	Q9JLU4	Shank3 [Proline-rich synapse associated protein 2] [21]	172.77	4.844	Plasma membrane; cytoplasm, synapse	Widely expressed in brain

JS40001396	Q64548	Reticulon-1 [30]	85.81	3.637	Endoplasmic reticulum	Expressed predominantly in central and peripheral nervous system. isoform RTN1-B is restricted to particular neuronal types

JS40046849	Q6GMN2	Brain-specific angiogenesis inhibitor 1-associated protein 2 (BAI1A2) [27]	25.66	**∞**	Plasma membrane; cytoplasm, cell projection	Ubiquitous

JS40014656	O08816	Neural Wiskott-Aldrich syndrome protein (N-Wash) [22]	16.95	**∞**	Cytoskeleton; nucleus	NA

JS40028986	NP_001101576	Plexin-B2 [28]	14.73	0.553	Plasma membrane	NA

JS40004987	Q63198	Contactin-1 [Neural cell surface protein F3 [23]	7.97	**∞**	Plasma membrane	NA

JS40001709	Q6RJR6	Reticulon 3 protein [26]	2	416.78	golgi; ER	many tissues

JS40039470	Q8NFP9	Neurobeachin [20]	1.85	**∞**	Peripheral membrane, cytoplasm	Predominant in many brain structures. Also in many other tissues

JS40011947	P21263	Nestin [25]	1.17	**∞**	unknown	CNS stem cell

Protein listed were taken from our rat lung EC PM proteome study published previously [2]. From left to right, columns contain: Avatar ID, the accession number from the RefSeq database, description of the protein, abundance, P/H ratio, annotated subcellular location, and tissue specificity according to UniProt and GO knowledge databases.

**Figure 10:**
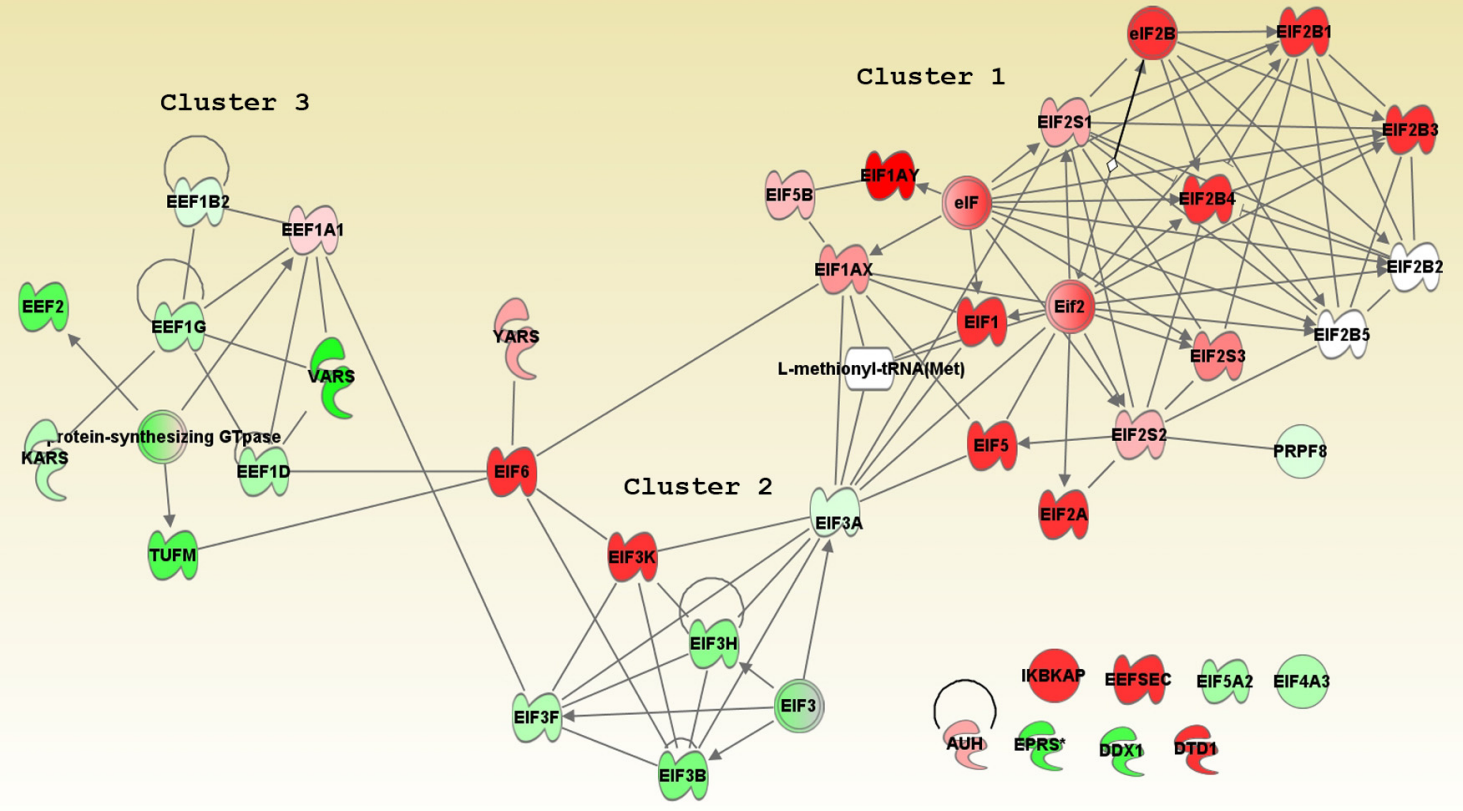
**Network of translation initiation and elongation factors**. Molecules in this network were derived from table 2. Only direct protein-protein interactions were allowed in the analysis. See figure 5 for the node colors and shapes.

As the regulation of translation initiation showed significant enrichment in the ontology analysis, we imported all molecules from table 2 into IPA to investigate the relationship among transcription and elongation factors in our membranome. All molecules in Table 2, except for aminoacyl-tRNA synthetase, were found in the IPAKDB. In total, 107 relationships were established by 36 nodes (Figure 10). Eight proteins (see Materials and Method); showed no interaction with the others in the network. Noticeably, this map was clustered into three individual groups: cluster 1 was formed by molecules highly enriched in P and showed stronger relationships with each other (20 nodes connected by 68 edges), whereas cluster 2 and 3 were formed by the molecules depleted in our P and showed weaker interactions with each other (16 nodes and 32 edges across both clusters). Cluster 1 and Cluster 2 were linked by EIF3A, while Cluster 1 and Cluster 3 were linked by EIF6. Together with the differential abundance and P/H ratio listed in Table 2 and the distinct relationship among these molecules, molecules in Cluster 1 are likely to exist at the PM and may be involved in local protein synthesis.

## Discussion

Tremendous effort has been placed on the discovery of biomarkers in the PM for the development of new drugs using a variety of technologies, particularly MS-based large-scale proteomics. Depending on the approach, between 100 to 1300 PM proteins have been identified with different types of mass spectrometers [31], ionization techniques [32], temperatures of C_18 _column [33], and protein separation strategies [32,34]. To date, we have provided the most comprehensive analysis of a PM proteome (over 1800 proteins) by using multiple techniques with replicate measurements to reach ~95% analytical completeness for each technique [2,3].

Here, we analyzed the functions of our EC PM proteome at the molecular and systems biology levels to further our understanding and assessment of this proteome. As expected, we identified many characteristic PM functions greatly overrepresented in this proteome, such as signaling, transport, adhesion, membrane docking, membrane budding and fusion, endocytosis, and exocytosis. Moreover, various EC-associated functions were also significantly enriched, including blood vessel development and maturation, angiogenesis, and negative regulation of EC proliferation.

As stated above, ECs exhibit differential protein expression in organs to perform distinct organ-specific functions [12]. The lungs are strategically positioned and specialized to metabolize a number of hormones circulating in the blood, usually to regulate blood pressure [8,9]. Unfortunately, current database curation does not extend sufficiently to document such rather lung-selective functions and processes. We did, however, detect overrepresentation of peptidase activity in our analysis. We found key peptidases well-known for mediating critical and basic lung functions, such as angiotensin converting enzyme, endothelin converting enzyme and aminopeptidase P, highly enriched in our dataset [2,10,11]. These enzymes are critical for regulating peptide hormones such as bradykinin, renin, and endothelin [8,9]. Here, our unbiased systems analysis did not readily detect apparent lung-specific metabolism of peptides and other lung-specific processes because the data is simply not parsed in this manner. Thus, it is clearly important to combine unbiased analysis with more targeted analysis of individual proteins to determine the true functions of EC surface proteins. These results provide basic functional information about this proteome and also confirm the proteins identified by our previous MS analysis as belonging to the EC PM.

Using IPA algorithms, we clearly demonstrated the formation of clusters among molecules assembled by their functions within the EC PM proteome, especially the networks currently not available in the public databases, for instance mechanotransduction/mechanosignaling. Since this category was created by our manual curation via database and article searches, revealing their relationship by IPA made it possible for us to prove the molecules in this category are indeed organized into a functional unit. Beyond that, we have discovered several new molecules that may also function in mechanotransduction and/or mechanosignaling (Figure 5a). Studying protein-protein interaction can also provide insight into the key roles of a general pathway like integrin-mediated signaling. Integrins, as a family of cell surface receptors, mediate numerous mechanical and chemical signals upon ligand binding, resulting in multiple and vital biological activities in a cell, such as cell cycle regulation, cell proliferation, cell differentiation, apoptosis, kinase activity, ion channel regulation, and organization of the actin cytoskeleton [35]. We also identified integrin-mediated cell motility and cytoskeletal interactions pathways at the center of integrin-mediated signaling pathways (Figure 6c). Thus, we can speculate that the mobility of the EC PM may be an essential and persistent function due to the potential of the PM to expand and sprout.

Our analysis indicates that some functions and processes associated with EC may be in common with neurons. We discovered several significantly enriched pathways that are generally thought of as neuronal functions, including neurotransmitter metabolic process (Figure 1d) and growth of neurites (Figure 4). Though initially surprising, the presence of neuron-associated proteins in the EC proteome is unlikely to be the result of contamination. EC and neuronal cells are distinct cell populations and there is no reason to detect neuronal membranes in the EC PM membrane preparation. Additionally, only a subset of neuronal proteins were found in our data set, and many high abundance neuronal proteins were missing, which indicates the lack of general contamination. Furthermore, a growing body of literature suggests that ECs and neurons may share many processes. ECs are known to express some classes of nicotinic non-neuronal acetylcholine receptors [4] and can synthesize and release the classic neurotransmitter acetylcholine [5] which can then act in an autocrine or paracrine fashion. Additionally, recent studies suggest that similar molecules and signaling pathways may act to pattern the nervous system and the vascular tree. Ephrins, semaphorins, slits, and netrins, along with their receptors, play well-known roles in axonal guidance. Many of these molecules may also provide guidance for EC during angiogenesis, blood vessel sprouting, and overall blood vessel patterning [36]. Similarly, very recent work suggests that VEGF receptor type 2, a molecule that plays a central role in EC proliferation, growth, and angiogenesis, can also be expressed by neurons and may participate in axonal pathfinding [37]. Thus, it is not unusual that proteins involved in growth of neurites and growth of EC are common to these two cell populations, as EC and neuronal cells share many cellular and molecular functions.

As shown in table 4, we detected several neuron-specific molecules in the EC PM membranome. Some of these proteins are now known to be expressed rather ubiquitously instead of restricted to neuronal cells, such as VAMPs [19], BAI1A2 [27], reticulon 3 [26], and neurobeachin [20]. Other proteins, such as neuropilin [29] and plexin B2 [28], are clearly found in both ECs and neuronal cells. Seven proteins (50%) in this list still remain as neuron- or brain-restricted proteins based on current knowledge, including N-catenin [24], reticulon 1&4 [18,30], shank 3 [21], N-wash [22], contactin 1 [23], and nestin [25]. Further work will be required to determine whether a different isoform exists in the EC PM. Regardless, the presence of neuron-specific proteins or protein families in our EC PM suggests clearly that PM or EC PM can perform functions similar to neurons.

Another unexpected finding in this work is the detection of translation as one of the highly enriched categories in our membranome. In principal, protein synthesis is located in the cytoplasm and the ER. Protein synthesis starts with the translocation of mRNA from the nucleus to the cytosol and ends with transport of newly synthesized proteins to their final subcellular destinations via ER and Golgi networks. However, more recent studies show that translocation and protein synthesis can occur at multiple subcellular locations, including the PM. Additionally, the location of protein synthesis often correlates with mRNA location [38-42]. It has been proposed that local protein synthesis can save energy for transporting the newly synthesized proteins and also permits rapid and efficient local responses [43]. Local protein translation in neuronal distal axons is well-accepted [review: [44]]. Neurons and EC can be highly attenuated; thus, local protein synthesis may occur in both cell types to support cell regions far from the nucleus. Further validation of this role at the PM requires identification of the machinery for synthesizing specific proteins.

We found that the regulation of translation initiation in response to stress was one of the two actual enriched functions in translation (Figure 3a). Russo and his coworkers identified a heat-induced secretory protein in yeast, hsp150 [45], that appears to be covalently attached to the cell wall, is involved in cell growth, cell shape, and cell wall strength, and plays a protective role during stress adaptation [46-50]. Thus, certain proteins in the heat shock family may reside and actually be synthesized at the PM in order to rapidly respond to changes in the surrounding environment.

In the study of local synthesis of focal adhesion proteins at cell junctions, Nilsson and his colleagues discovered a protein, RACK1 (receptor for activated C-kinase), bound to the integrin-β receptor, ribosomes, and PKC at the PM. They also found RACK1 interacting with talin and vinculin, two important focal-adhesion molecules [51]. RACK1 was first found to exist in an early stage of the spreading initiation centers (SIC) and regulated translation of specific mRNAs in yeast. SIC are ribonucleoprotein complexes containing focal adhesion markers, numerous RNA binding proteins, ribosomal RNA, and perhaps other RNAs to play a role in the initiation of cell spreading [52]. This suggests that focal-adhesion molecules may be synthesized at the PM via binding of RACK1 to membrane receptors, primarily to promote ribosome docking at the site where new proteins are required. In addition, Klein *et al*. reported co-localization of eIF2 with GPCRs at the PM [53], and Bassell and Singer showed that many elongation factors and helicases associated into multi-protein complexes to translate RNA into proteins. These complexes can also associate with the PM through their connections with the cytoskeleton [54].

We detected RACK1, a number of RNA binding proteins, ribosomal proteins, and eIFs in our membranome. Interestingly, we also detected one of the best-studied neuronal mRNA transport protein, staufen, a dsRNA binding protein. Staufen plays a role in regulation of the dendritic cytoskeleton. Its deficiency in neurons caused severe deficits in dendritic spine morphology, size, and density, which might be due to dysfunction in the delivery of β-actin mRNA [55]. In view of Staufen found in our proteome, it may be sensible to determine whether β-actin can be synthesized at the EC PM. Importantly, we confirmed the presence of several protein synthesis-related proteins in our EC PM membranome and at the EC surface of blood vessels by immunoblotting and IHC (Figure 3b and 3c).

We and others have identified TyrRS association with the EC PM. In 1999, Wakasugi and Schimmel discovered that TyrRS is secreted into the EC extracellular matrix under apoptotic conditions [56]. They showed that TyrRS is cleaved into two fragments upon secretion, and its N-terminal fragment, mini TryRS, is an angiogenic factor [57]. More recently, mini TyrRS (43 KDa) was found endogenously present in ECs, and could be secreted upon treatment of the intact ECs with TNF-α [58]. These studies support our speculation that TyrRS can be PM-associated, but question our suggestion that protein synthesis can occur at the PM. We did not detect the truncated mini TyrRS protein in P; both full length and truncated TyrRS were detected in H (Figure 3b). Plus, the 59 KDa band showed substantially stronger signal than the 43 KDa band. Thus, we can conclude that the TyrRS in this membranome existed as the full length protein which plays a role in protein synthesis rather than cytokine signaling of the mini TyrRS. Noticeably, two bands with equal signal intensity around 59 KDa were detected in our P only by immunoblotting, indicating a PM-specific isoform of TyrRS may be present in our sample.

We were a little surprised at first when we detected angiogenesis as an overrepresented function in the EC PM, given the quiescent nature of ECs under normal physiological conditions. Expanding this category revealed that the molecules playing major roles in this category all function in negative regulation of angiogenesis, wound healing, and patterning blood vessels. These functions should be present in normal blood vessels, probably to retain the structure and function of the mature blood vessels. Patterning blood vessel describes a process that regulates the coordinated growth and sprouting of blood vessels leading to the organized vascular system. Its enrichment in normal blood vessels raises concerns regarding certain cancer therapeutics. Angiogenesis has been well defined as a fundamental step in the transition of tumors from a dormant state to a malignant state, which is the foundation for development of many anti-angiogenic cancer therapeutic drugs. The finding here in normal lung vasculature implies that certain angiostatic drugs may inhibit essential normal blood vessel development, which should be taken into consideration in future drug testing and discovery and may help explain recurrent vascular side effects.

Our laboratory has pioneered the isolation of luminal EC PM directly from tissues *in vivo *[2,10-12,59-61]. Here, we demonstrate the power of combining proteomic mapping with systems analysis to define functions attributable to these PM. It is likely that further technology development will continue to expand the EC PM and reveal new proteins and functions at this vital interface. Our extensive analysis revealed most functions were well known to be associated with PM in general and with the EC surface in particular. Many researchers have devoted decades to establish PM and EC surface functions by defining one-by-one the specific role of each protein. Because ECs can be difficult to study *in vivo*, investigators were forced to focus primarily on ECs *in vitro *after isolation and growth in cell culture, both of which can contribute to extensive changes in protein expression [10]. Here, we rapidly designate PM- and EC PM-associated functions on membranes isolated from EC *in vivo*. This analysis helps to validate the quality of the isolated EC PM and shows that they do indeed encompass EC surface functions. We have, for the first time using a large-scale systems approach, also identified a significant cross-relationship between ECs and neuronal cells by showing that ECs express key neuron-restricted proteins *in vivo*. In conclusion, the combination of large-scale proteomic mapping with systems analysis provides a robust and unbiased strategy to define functions of the PM and vascular endothelium under native condition in tissue *in vivo*.

## Materials and methods

### Data used in this study

The protein list used in this work was determined in a large-scale MS-based proteomic analysis described in our earlier report [2]. These proteins were identified from EC PM isolated from rat lung vasculature *in vivo *using a nanoparticle-based subcellular fractionation of lung tissue [60,61] and multi-dimensional protein and peptide separation with mass spectrometry [2], both technologies developed and optimized in our lab.

### Relative protein enrichment in membrane

The relative enrichment of proteins was determined by the ratio of the average SI_N _value for a given protein identified in membranes (P) to the average SI_N _value of the same protein found in entire tissue homogenates (H) (P/H ratio). SI_N _is a normalized label-free quantification method developed in our lab recently [3]. The proteins with a value of P/H >1.0 were defined as PM-enriched proteins, whereas proteins with P/H < 1.0 were depleted.

### Functional annotation and enrichment analysis

Biological process and molecular function, when known, were assigned to each protein based on Panther http://www.pantherdb.org/[62,63], UniProt http://www.uniprot.org/uniprot[64] FatiGO http://babelomics.bioinfo.cipf.es, and Gene Ontology annotations (GO, http://www.geneontology.org). The category of mechanotransduction/mechanosignaling is a well-defined PM-associated function. As this function is absent from the selected public databases, we created it by manually importing relevant proteins into our in-house protein database, Avatar, curated via articles and public databases searches. Proteins with multiple molecular functions and biological processes were included in multiple categories. Each annotated function was assigned a Z-score to measure if a given function or process was significantly over or underrepresented in our EC PM proteome relative to the public databases (see Eq. 1).(1)

*a = *the number of proteins in a category observed in our EC PM membranome;

*n = *the total number of proteins observed in our EC PM membranome;

*A = *the number of proteins in a category in public database;

*N = *the total number of proteins in public database.

As described in Eq. 1, the proportional enrichment or depletion of a function or process in this membranome was determined by the number of proteins in our dataset in comparison with the number in the selected public database of the same category. Positive Z-scores indicated relative overrepresentation or enrichment, whereas negative scores indicated relative underrepresentation or depletion. We assumed a hypergeometric distribution; therefore, Z-scores ≥2.0 or ≤-2.0 [~95% Confidence Interval (CI)] was considered significantly enriched or depleted, respectively.

### Protein-protein interactions and pathway analysis

We used IPA algorithms to define all possible protein-protein interaction networks in the EC PM membranome based on the IPA Knowledge Database (IPAKDB, Ingenuity^® ^Systems, http://www.ingenuity.com). The rank of strength of each network was determined and grouped by its IPA score, which is the negative log of its p-value of hypergeometric distribution. This score is calculated with the right-tailed Fisher's Exact Test, which takes into account the number of eligible molecules in a network, the size of the network, and the total number of molecules known to be associated with that network in IPAKDB http://www.ingenuity.com. In general, the more molecules found in our dataset for a given pathway, the higher the score will be for this pathway. The p-value of each network was also taken from IPA. It considers the number of molecules participating in that function and the total number of molecules that are known to be associated with the function in IPAKDB. The smaller the p-value is, the less likely that the network is random. P-values < 0.05 indicate a statistically significant, non-random network http://www.ingenuity.com.

### Immunoblotting

CA150 and SAP130 antibodies were kindly supplied by Drs. Garcia-Blanco (Department of Molecular Cancer Biology, Duke University Medical Center) and Reed (Department of Cell Biology, Harvard Medical School), respectively. Antibodies against tyrosyl-tRNA synthetase was purchased from Abcam (Cambridge, MA); antibodies against eukaryotic translation initiation factor 1 gamma and eukaryotic translation initiation factor 3 subunit 9 were purchased from Aviva Systems Biology (San Diego, CA). Western blot analysis was performed as per standard protocols described previously [65].

### Electron microscopy

Rat lungs were flushed with tissue culture medium and perfused with 4% paraformaldehyde in situ. Then the tissue was cut into ~1 mm^3 ^pieces and incubated for 2 hrs at 4°C in the same fixative. Following fixation, tissue was vitrified by infiltrating the pieces with 50% polyvinylpyrrolidone containing 2.3 M sucrose in 0.1 M phosphate buffer, pH 7.4, for 1-2 hrs or overnight. Immunocytochemical staining was performed as described previously [66]. In brief, 60 nm sections of frozen rat lung tissue were cut with a diamond cryo 35° knife (Diatome) on an EM FC-S low temperature sectioning system (Leica). Sections were picked from the knife with 2.3 M sucrose, transferred to formvar-carbon-coated nickel grids, and floated on 1% albumin of chicken egg white (Sigma, Cat No.A5378) in 0.1 M Na-cacodylate buffer for at one hour before incubation with antibody against tyrosyl-tRNA synthetase (10 - 150 μg/ml), antibody against contactin 1 (50 μg/ml, R&D systems, MN), or antibody against neuropilin 1 (50 μg/ml, Santa Cruz Biotechnology Inc., CA) at room temperature for one hour. Sections were then washed eight times with 0.1% ovalbumin in the same buffer and incubated with 10 nm Au coupled to protein A (Cell Microscopy Center, University Medical Center Utrecht, Netherlands). After washing with the same buffer, the labeled section was fixed with 1% glutaraldehyde and stained with a mixture of uranyl acetate and methyl cellulose (25 centipoises, Sigma M-6385) in water at final concentration of 1.3% each for 10 min at room temperature. Images were obtained with Morgagni 268 D electron microscope equipped with MegaView III digital camera at 100 kV, magnification of 71 K.

## Competing interests

The authors declare that they have no competing interests.

## Authors' contributions

YL produced the list of proteins, conducted all data analyses, and generated this manuscript; KM provided neuron-associated information and edited this manuscript; HW provided EM data; and JS supervised all aspects of this manuscript. All authors read and approved the final manuscript.

## Supplementary Material

Additional file 1 **Figure S1. Expanded general biological processes of cellular component organization**. All functional terms in the GO rat knowledge database http://www.geneontology.org were analyzed to uncover all subcategories revealing cellular component organization which linked to final actual enriched function. Only the terms directly relating to the final function were expanded in each layer (highlighted in red between grouped bars). The over-or underrepresentation of a category was determined by a Z-score ≥ 2 or ≤ -2, respectively, shown as dotted lines. Charts are arranged with the top level category at the bottom and resident subcategories above it. **Figure S2. Expanded general biological processes of localization**. All functional terms in the GO rat knowledge database http://www.geneontology.org were analyzed to uncover all subcategories revealing localization which led to the final actual enriched function. Only the terms directly relating to the final function were expanded in each layer (highlighted in red between grouped bars). See figure S1 for detailed information for the chart. **Figure S3. Expanded general biological processes of metabolic process**. All functional terms in the GO rat knowledge database http://www.geneontology.org were analyzed to uncover all subcategories revealing metabolic process as an enriched function. **: cellular macromolecule metabolic process has been expanded in Figure 3a. See figure S1 for detailed information for the chart. **Figure S4. Expanded general biological processes of cellular process**. All functional terms in the GO rat knowledge database http://www.geneontology.org were analyzed to uncover all subcategories, revealing cellular process as an enriched function. Only the terms directly relating to the final function were expanded in each layer. See figure S1 for detailed information for the chart. **Figure S5. Expanded general biological processes of developmental process**. All functional terms in the GO rat knowledge database http://www.geneontology.org were analyzed to uncover all subcategories, revealing developmental process as an enriched function. Only the terms directly relating to the final function were expanded in each layer. See figure S1 for detailed information for the chart. **Figure S6. Expanded general molecular function of binding**. All functional terms in the GO rat knowledge database http://www.geneontology.org were analyzed to uncover all subcategories, revealing binding as an enriched function. Only the terms directly relating to the final function were expanded in each layer. See figure S1 for detailed information for the chart. **Figure S7. Expanded general molecular function of catalytic activity**. All functional terms in the GO rat knowledge database http://www.geneontology.org were analyzed to uncover all subcategories, revealing catalytic activity as an enriched function. Only the terms directly relating to the final function were expanded in each layer. See figure S1 for detailed information for the chart. **Figure S8. Expanded general molecular function of transporter activity**. All functional terms in the GO rat knowledge database http://www.geneontology.org were analyzed to uncover all subcategories, revealing transporter activity as an enriched function. Only the terms directly relating to the final function were expanded in each layer. See figure S1 for detailed information for the chart. Click here for file
