# Supplementary figures and images for: Systems analysis of endothelial cell plasma membrane proteome of rat lung microvasculature
Source: Proteome Sci. 2011 Mar 29;9:15. doi: 10.1186/1477-5956-9-15 (PMC3080792; doi:10.1186/1477-5956-9-15)

Figure S1.

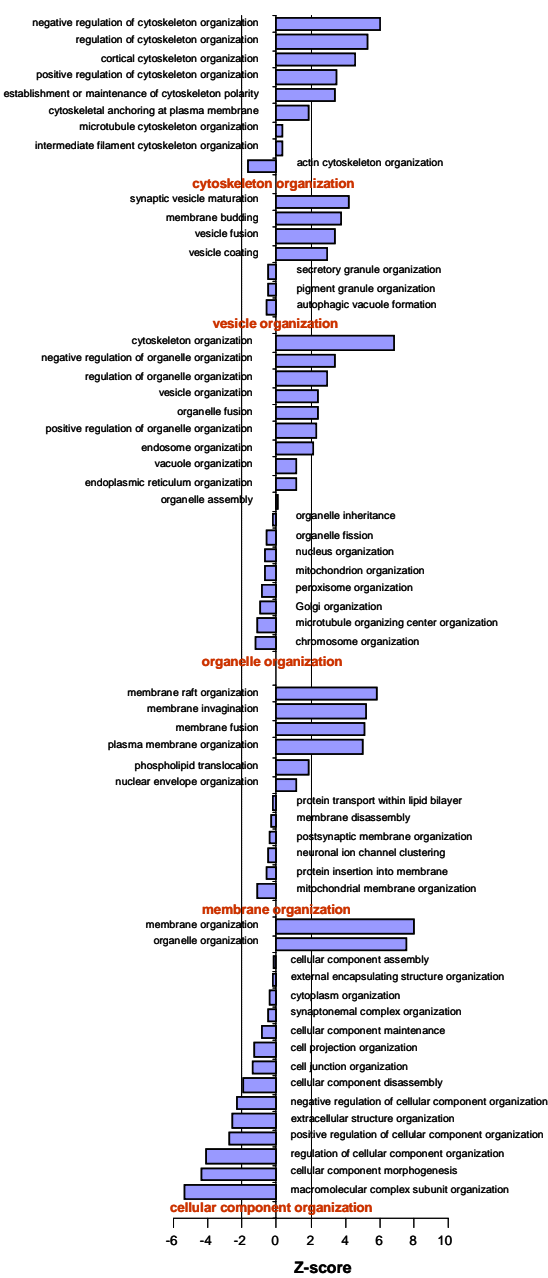

Figure S2.

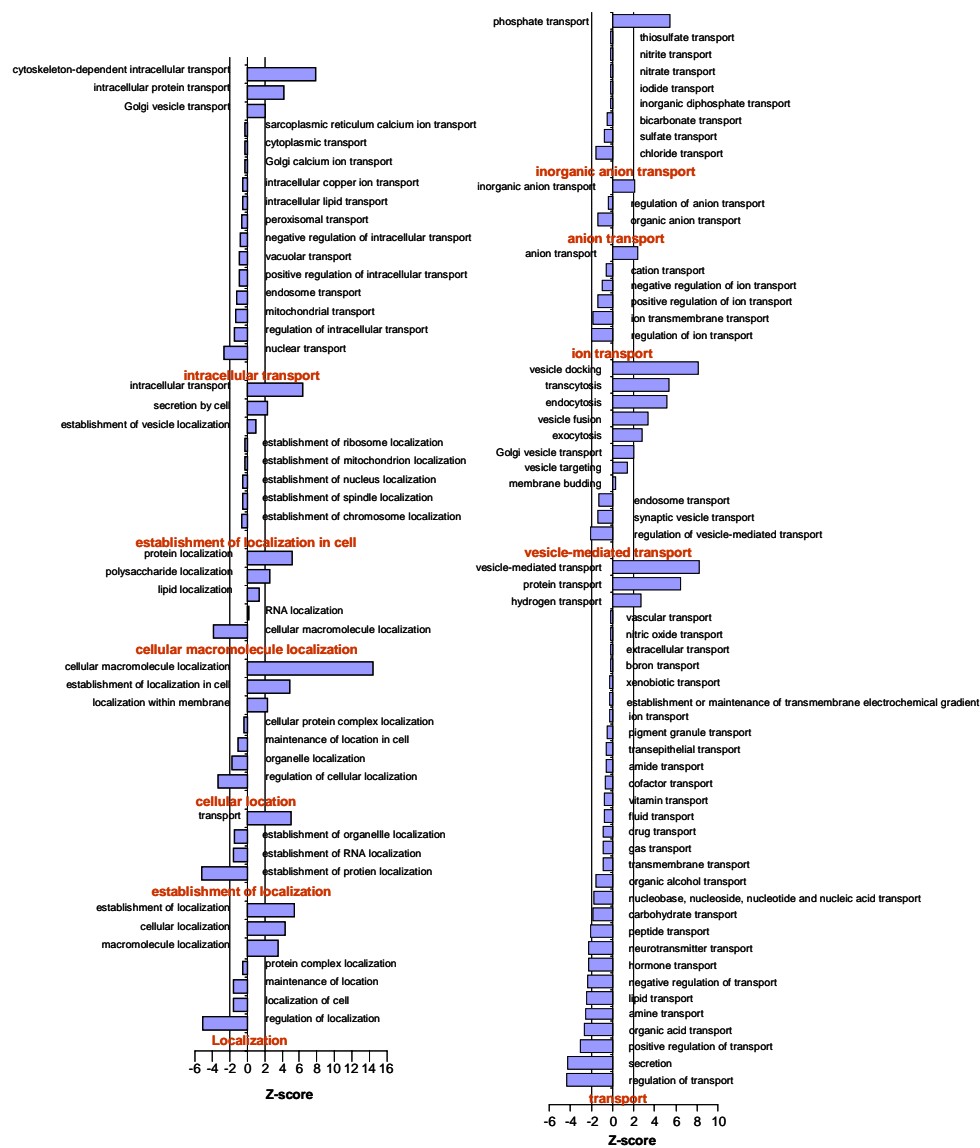

Figure S3.

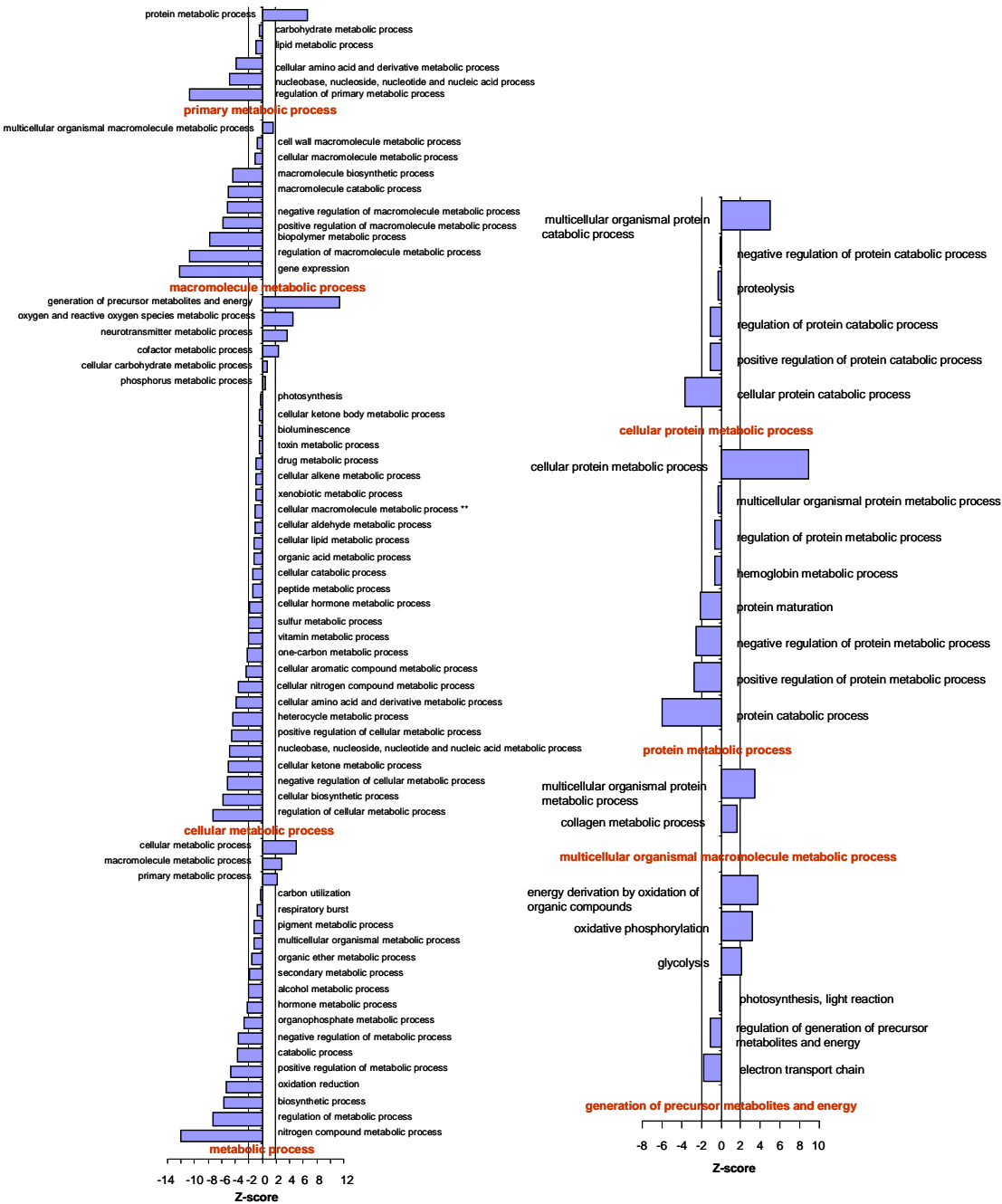

Figure S4.

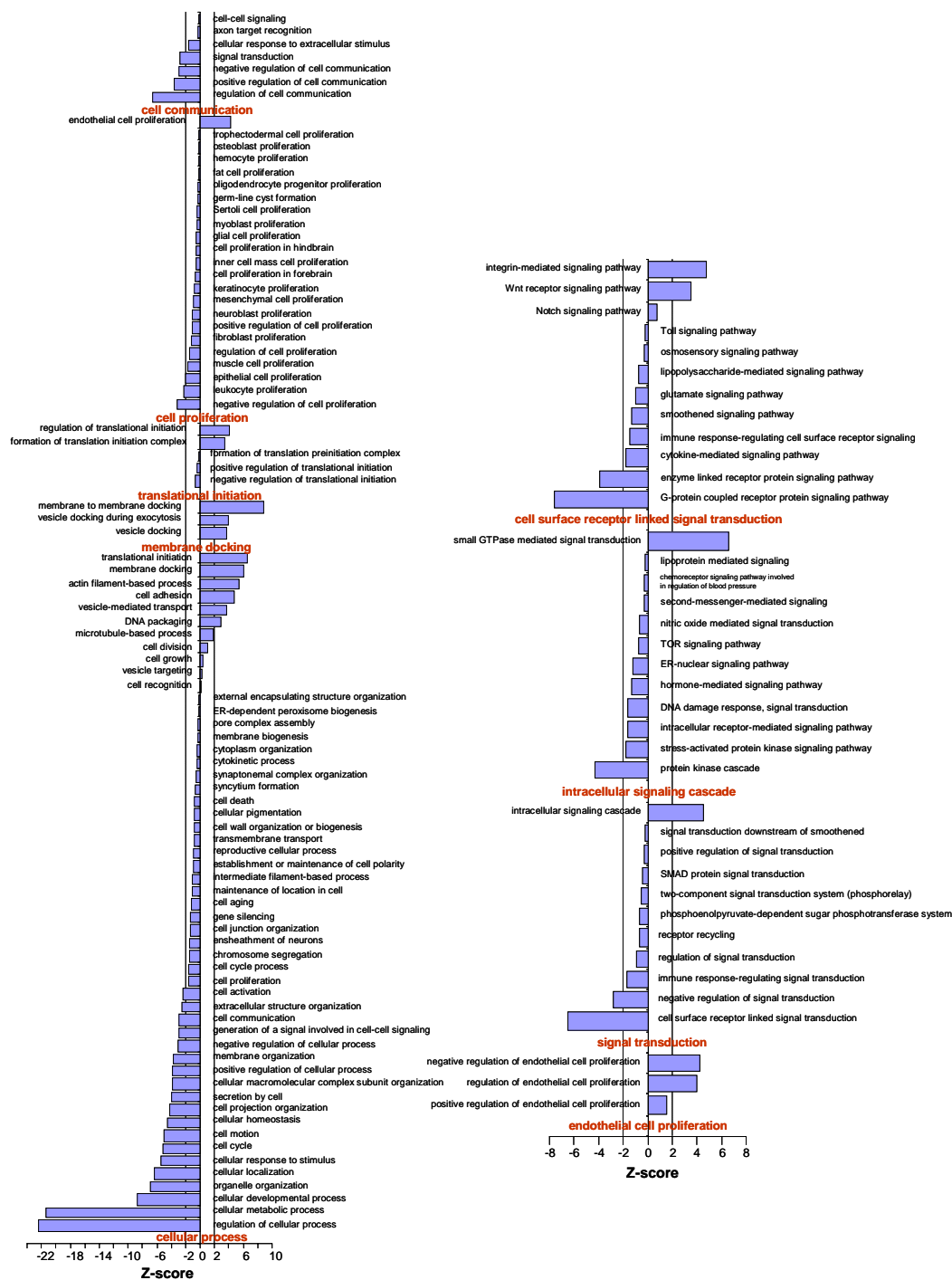

Figure S5.

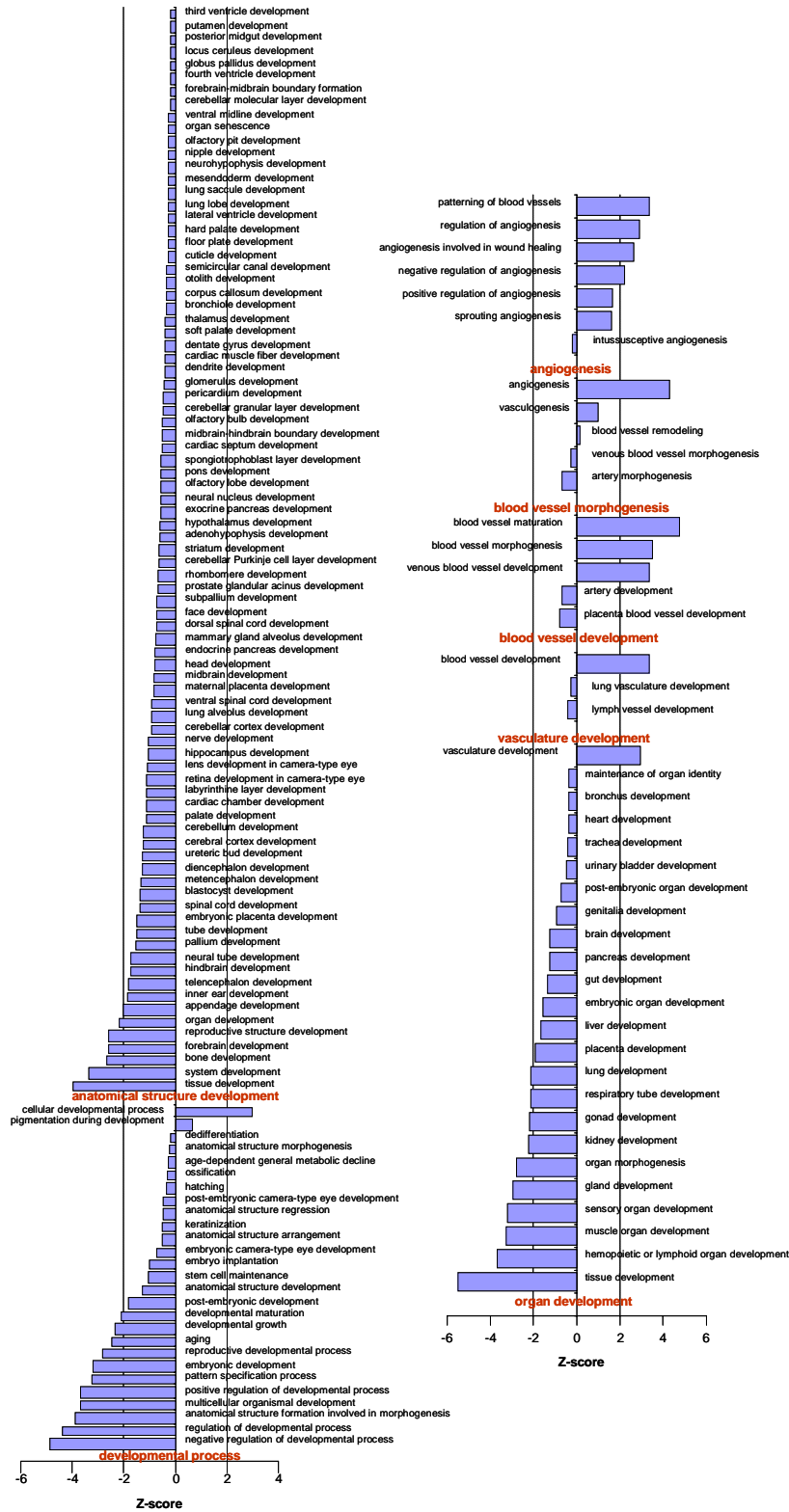

Figure S6.

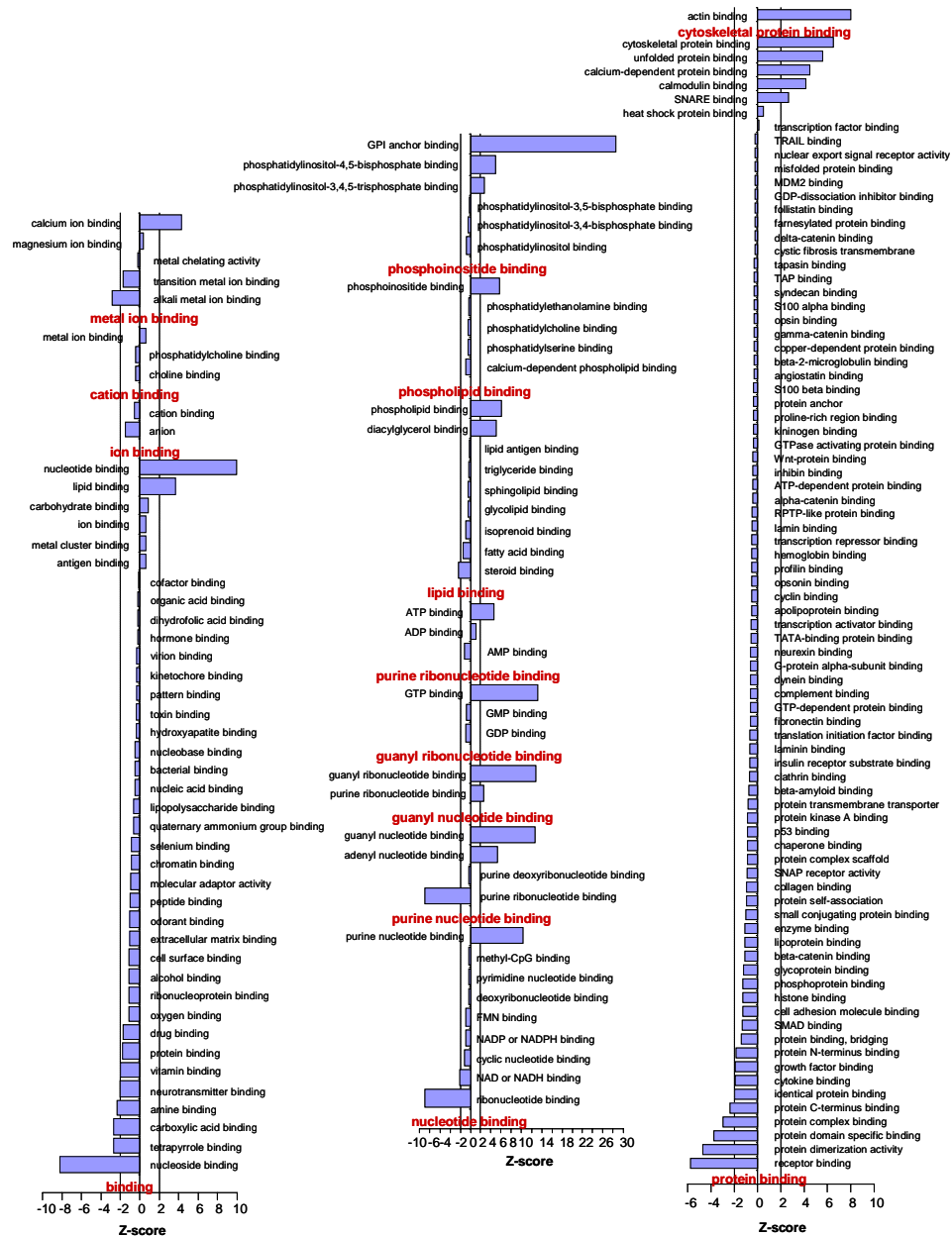

Figure S7.

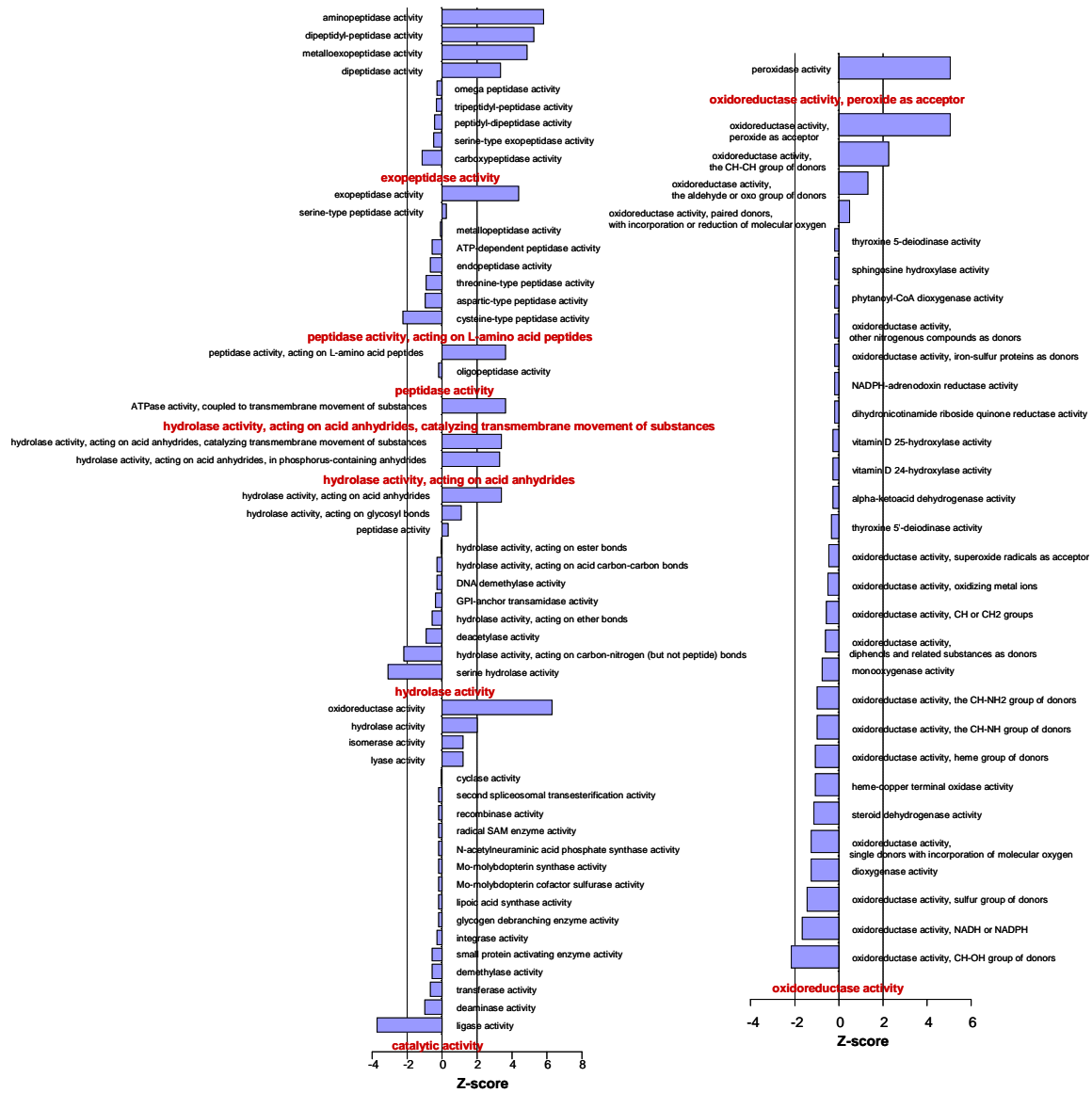

Figure S8.

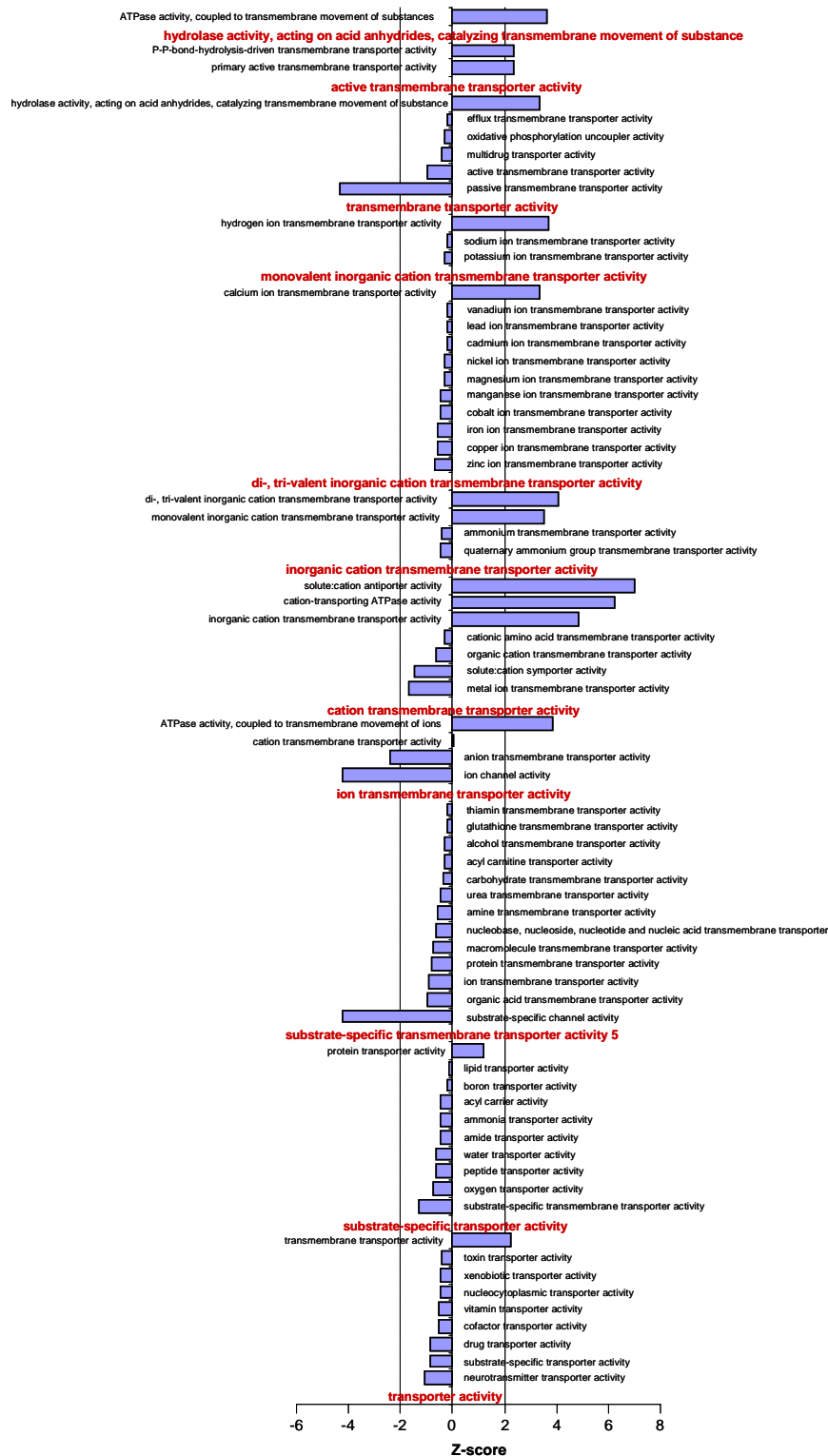

Supplement: Additional file 1 — Figure S1. Expanded general biological processes of cellular component organization. All functional terms in the GO rat knowledge database http://www.geneontology.org were analyzed to uncover all subcategories revealing cellular component organization which linked to final actual enriched function. Only the terms directly relating to the final function were expanded in each layer (highlighted in red between grouped bars). The over-or underrepresentation of a category was determined by a Z-score ≥ 2 or ≤ -2, respectively, shown as dotted lines. Charts are arranged with the top level category at the bottom and resident subcategories above it. Figure S2. Expanded general biological processes of localization. All functional terms in the GO rat knowledge database http://www.geneontology.org were analyzed to uncover all subcategories revealing localization which led to the final actual enriched function. Only the terms directly relating to the final function were expanded in each layer (highlighted in red between grouped bars). See figure S1 for detailed information for the chart. Figure S3. Expanded general biological processes of metabolic process. All functional terms in the GO rat knowledge database http://www.geneontology.org were analyzed to uncover all subcategories revealing metabolic process as an enriched function. **: cellular macromolecule metabolic process has been expanded in Figure 3a. See figure S1 for detailed information for the chart. Figure S4. Expanded general biological processes of cellular process. All functional terms in the GO rat knowledge database http://www.geneontology.org were analyzed to uncover all subcategories, revealing cellular process as an enriched function. Only the terms directly relating to the final function were expanded in each layer. See figure S1 for detailed information for the chart. Figure S5. Expanded general biological processes of developmental process. All functional terms in the GO rat knowledge database http://www.ge [file 1477-5956-9-15-S1.PDF]
